# A kinetic ruler controls mRNA poly(A) tail length

**DOI:** 10.1101/gad.352912.125

**Published:** 2025-11-01

**Authors:** Emilie Gabs, Emil Aalto-Setälä, Aada Välisaari, Anssi M. Malinen, Torben Heick Jensen, Stephen H. McLaughlin, Lori A. Passmore, Matti Turtola

**Affiliations:** 1 Department of Life Technologies, University of Turku, Turku 20520, Finland;; 2 Department of Molecular Biology and Genetics, Aarhus University, Aarhus 8000, Denmark;; 3 Laboratory of Molecular Biology, Cambridge CB2 0QH, United Kingdom

**Keywords:** CCCH zinc finger protein, cleavage and polyadenylation complex (CPAC), kinetic ruler, mRNA polyadenylation, Nab2, poly(A) binding protein (PABP), poly(A) tail, RNA-binding protein, ZC3H14

## Abstract

Poly(A) tails of newly synthesized mRNAs have uniform lengths, arising through cooperation between the cleavage and polyadenylation complex (CPAC) and poly(A) binding proteins (PABPs). In the budding yeast *Saccharomyces cerevisiae*, the responsible PABP is the evolutionarily conserved CCCH zinc finger protein Nab2 that facilitates the biogenesis of ∼60 adenosine mRNA poly(A) tails. Here, we address the molecular basis for such length control. Reconstituting polyadenylation reactions during the formation of Nab2:poly(A) RNA ribonucleoprotein particles in vitro, we found that Nab2 dimerization directs polyadenylation termination. The Nab2 dimer is stable only on poly(A) tails that are >25 adenosines, explaining how Nab2 avoids prematurely terminating poly(A) synthesis. However, the mature tail length is not determined by the footprint of Nab2 on the RNA but rather by the kinetic competition between CPAC-mediated tail elongation and Nab2 RNA binding. Variations in Nab2 RNA binding rate can shift poly(A) tail lengths, but in cells such variations are buffered by autoregulation of Nab2 protein concentration. As a result, poly(A) tail length control operates through a “kinetic ruler” mechanism, whereby the concentration of Nab2 quantifies RNA length.

## Abstract (precis)

In this study, Gabs et al. show that poly(A) tail length is dictated by kinetic competition between poly(A) tail elongation mediated by the cleavage and polyadenylation complex and polyadenylation termination directed by the zinc finger poly(A) binding protein NAB2 in *Saccharomyces*. NAB2 dimerization and multidomain RNA binding are counterbalanced by the autoregulation of NAB2 protein concentration, which together fine-tune mRNA poly(A) tail synthesis and thus mRNA stability.

RNA 3′ end polyadenosine [poly(A)] tails affect all main post-transcriptional steps of gene expression. A significant property of the poly(A) tail is its length, which impacts the recruitment of poly(A) binding proteins (PABPs) and is functionally linked to export, translatability, and stability of the RNA (Passmore and Coller 2022). Poly(A) tails of mRNAs are synthesized by the multisubunit cleavage and polyadenylation complex (CPAC), and because the reaction is nontemplated, the product length must be controlled by auxiliary factors, which are largely PABPs (for reviews, see Eckmann et al. 2011; Boreikaite and Passmore 2023; Rodríguez-Molina and Turtola 2023). As a result, all polyadenylated mRNAs are initially produced with a species-characteristic tail length, with newly synthesized yeast and mammalian poly(A) tails having ∼60 and ∼250 adenosines (As), respectively.

From its uniform starting point, the length of the poly(A) tail of a given mRNA varies throughout its life cycle as a result of nuclear and cytoplasmic activities that either extend or shorten the tail (Eckmann et al. 2011; Lim et al. 2016; Eisen et al. 2020; Tudek et al. 2021; Passmore and Coller 2022; Alles et al. 2023; Xiang et al. 2024). Thus, the steady-state poly(A) tail profile reflects its different cellular phases. Poly(A) tail length control provides a uniform entry point for deadenylases that gradually shorten the poly(A) tail with variable, transcript-specific, and condition-specific rates (Passmore and Coller 2022). Deadenylation generally stimulates full mRNA degradation (Parker 2012; Webster et al. 2018; Eisen et al. 2020; Czarnocka-Cieciura et al. 2024), often by promoting decapping, which can also be a rate-limiting step (Wiener et al. 2021; Audebert et al. 2024). Initial poly(A) tail length is therefore a key parameter in determining mRNA abundance.

In *Saccharomyces cerevisiae*, the nuclear PABP Nab2 is the primary factor controlling mRNA poly(A) tail length. It does so by associating with the growing poly(A) tail and inhibiting the polyadenylation activity of the CPAC (Hector et al. 2002; Viphakone et al. 2008; Turtola et al. 2021). Nab2 binding is further coupled to the formation of an export-competent messenger ribonucleoprotein particle (mRNP), as Nab2 interacts with proteins required for mRNP export (Carmody et al. 2010; Iglesias et al. 2010; Asada et al. 2023; Bonneau et al. 2023), facilitates mRNP docking to the nuclear pore complex (Fasken et al. 2008; Saroufim et al. 2015), and, during its residence in the nucleus, protects the newly synthesized transcript from degradation (Schmid et al. 2015; Tudek et al. 2018; Turtola et al. 2021). Conversely, errors in mRNP assembly or export disrupt Nab2-mediated poly(A) tail length control and lead to rapid degradation of misprocessed transcripts (Hilleren and Parker 2001; Jensen et al. 2001; Libri et al. 2002; Rougemaille et al. 2007; Tudek et al. 2018; Turtola et al. 2021). Consequently, Nab2 function is required for the proper production of RNA polymerase II (Pol II) transcribed mRNAs (González-Aguilera et al. 2011; Schmid et al. 2015).

The Nab2 homologous protein in animals, ZC3H14, has been reported to associate with mRNP biogenesis factors and components of the spliceosome (Soucek et al. 2016; Morris and Corbett 2018; Li et al. 2024), impact alternative splicing (Jalloh et al. 2023), and promote circular RNA generation through backsplicing (Li et al. 2024). Moreover, ZC3H14 has been implicated in the surveillance of noncoding RNAs (Latour et al. 2025) and the turnover of prematurely terminated Pol II transcripts (Insco et al. 2023). However, depletion studies have also pointed to ZC3H14 restricting poly(A) tail length (Pak et al. 2011; Kelly et al. 2014; Bienkowski et al. 2017; Rha et al. 2017; Morris and Corbett 2018; Li et al. 2024). Loss of the ZC3H14 homolog dNab2 in *Drosophila* decreases viability and causes defective neuronal development (Pak et al. 2011; Kelly et al. 2014, 2016). Likewise, in mouse models, loss of functional *Zc3h14* impairs neuronal development (Rha et al. 2017) and disrupts spermatogenesis (Li et al. 2024), whereas loss-of-function mutations in human *ZC3H14* lead to intellectual disability (Pak et al. 2011). Finally, in *Caenorhabditis elegans* and mouse brains, the protein affects the susceptibility to pathological tau; hence, its alternative name, suppressor of tau 2 (SUT-2/MSUT2) (Guthrie et al. 2009; Wheeler et al. 2019). Altogether, these diverse phenotypes await mechanistic explanations as to how Nab2/ZC3H14 regulates different RNA processing activities. This might be more easily revealed in organisms such as *S. cerevisiae*, in which a limited set of nuclear PABPs simplifies the analysis of poly(A) tail-mediated regulation.

PABP-mediated regulation of enzymes that either extend or shorten poly(A) tails is tied to their length-dependent interaction with poly(A) RNA. The current picture of how this is achieved comes from studies on RNA recognition motif (RRM)-containing PABPs, including mammalian PABPN1 and PABPC. These PABPs align their RRM domains side by side along the poly(A) RNA chain, like beads on a string (Eckmann et al. 2011; Passmore and Coller 2022). Consequently, the RNA footprints of the RRMs (11 adenosines for PABPN1 [Meyer et al. 2002] and 27 adenosines for PABPC [Baer and Kornberg 1980]) translate into periodic molecular rulers that control enzyme processivities in poly(A) tail length-dependent manners. In the case of PABPN1, the assembly of 15–20 PABPN1 molecules on an ∼250 A long poly(A) tail collapses the RNP into a globular structure, which is suggested to terminate processive polyadenylation by disrupting the interactions between the poly(A) tail-bound PABPN1, the poly(A) polymerase (PAP), and the rest of the CPAC machinery (Kühn et al. 2009, 2017). On the other hand, the cooperation of the poly(A)-bound PABPC and exonuclease activities results in poly(A) tail shortening to lengths that have ∼30 A periodicity (Azoubel Lima et al. 2017; Webster et al. 2018; Schäfer et al. 2019). However, a similar structure–function relationship has not yet been established for structurally different Nab2/ZC3H14 PABPs, which belong to Cys–Cys–Cys–His (CCCH)-type zinc finger (ZnF) proteins.

Compared with RRM-type PABPs, the CCCH ZnF Nab2 has a profoundly different topology for interacting with poly(A) RNA. It displays a diffuse nuclease-protected poly(A) RNA footprint (Viphakone et al. 2008), harbors structurally nonsimilar tandem ZnF domains that mediate RNA binding (Brockmann et al. 2012; Martínez-Lumbreras et al. 2013), and uses an unusual mode of RNA interaction in which a dimeric interface between three ZnFs from two Nab2 molecules creates a binding surface for seven As (Aibara et al. 2017). The functional significance of this dimeric architecture remains elusive and is therefore at present insufficient to explain how Nab2/ZC3H14 proteins are able to specify mRNA poly(A) tail lengths.

Here, we examine the biochemical basis of how Nab2, the founding member of CCCH ZnF PABPs (Fasken et al. 2019), interacts with poly(A) RNA to regulate its tail length during CPAC-mediated polyadenylation reaction. We found that, although Nab2 dimerization and its multidomain RNA-binding mode sensitize its interaction to different poly(A) RNA lengths, its footprint on poly(A) RNA does not readily determine poly(A) tail lengths. Rather, such control relies on the finely tuned kinetics of Nab2:poly(A) binding that competes with the poly(A) tail synthesis by the CPAC. We propose that Nab2 acts as a concentration-dependent kinetic ruler that quantifies RNA chain length.

## Results

### Time-resolved measurements of poly(A) tail elongation by the CPAC reveal distinct reaction phases

**Figure 1.**
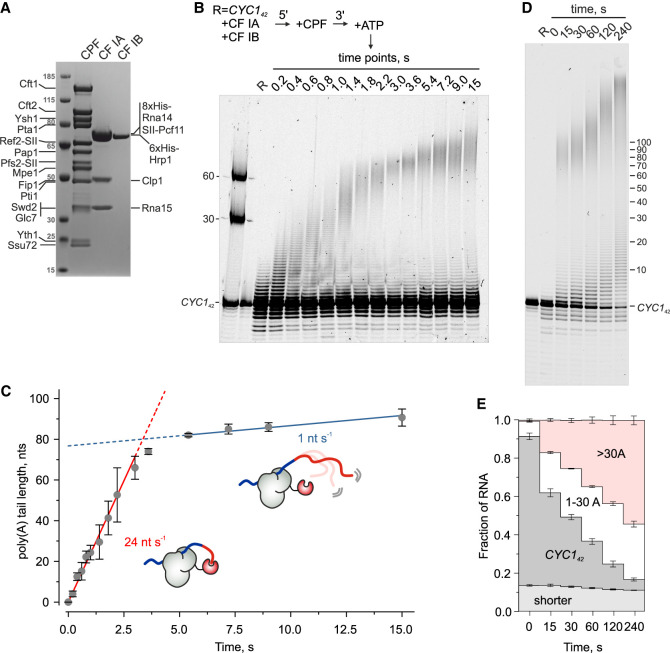
Time-resolved measurement of RNA polyadenylation by the CPAC. (*A*) SDS-PAGE of CPAC proteins used for reconstituting the polyadenylation reactions. (SII) Twin strep tag. (*B*) Time-resolved polyadenylation of 50 nM 5′Atto680-labeled *CYC1*_*42*_ RNA by the CPAC (50 nM CPF and 225 nM CF IA/IB). Reactions were initiated by mixing the preassembled CPAC with 2 mM ATP in a quench flow apparatus and stopped with HCl at the indicated times. Products were resolved by denaturing PAGE and visualized by fluorescence. Markers indicate poly(A) tail lengths. (*C*) Quantification of elongation kinetics. Mean poly(A) tail lengths (see the Materials and Methods) from three replicates are shown with SD. Elongation rates were determined from the linear fit to the first 10 time points for the initial 70 As (red line) and to the last four time points for elongation beyond 80 As (blue line). The schematics illustrate the proposed fast and slow modes of poly(A) tail elongation. (Gray) CPAC, (red) poly(A) polymerase. (*D*) Extended time course of *CYC1*_*42*_ RNA polyadenylation with the same reaction conditions as in *B*. (*E*) Quantification of RNA species from *D* as stacked bars (mean ± SD, *n* = 3). (Dark gray) Nonprocessed *CYC1*_*42*_, (white) *CYC1*_*42*_ with 1–30 A tail, (red) *CYC1*_*42*_ with >30 A tail, (light gray) cleavage products.

To understand Nab2-mediated poly(A) tail length control, we first characterized the kinetics of CPAC-mediated polyadenylation reactions. The CPAC was reconstituted using purified CPF, CF IA, and CF IB subcomplexes (Fig. 1A). As a substrate, we used 42 nt RNA from the *CYC1* 3′ UTR (*CYC1*_*42*_), 5′-labeled with Atto680. *CYC1*_*42*_ contained the CPF-recognized polyadenylation signal (PAS) sequence and flanking sequences bound by CF IA and CF IB (Dichtl and Keller 2001; Hill et al. 2019), whereas its 3′ end corresponded to the CPF cleavage site, making it a “precleaved” substrate for isolated polyadenylation reaction (Viphakone et al. 2008; Schmid et al. 2012).

Polyadenylation was initiated by ATP addition and stopped at defined time points using quench flow to resolve elongation kinetics at subsecond resolution. As shown in Figure 1B and quantified in Figure 1C, a fraction of *CYC1*_*42*_ RNA was elongated by ∼70 As with a rate of 24 nt sec^−1^. These adenosines were added in a single wave and in an apparently processive manner, indicating a continuous engagement of the CPF and the poly(A) polymerase Pap1. Because CF IA and CF IB are required for efficient polyadenylation (Gross and Moore 2001; Casañal et al. 2017; Turtola et al. 2021), the whole CPAC was evidently assembled on the RNA substrate. Notably, the rate of poly(A) tail elongation subsequently slowed down, with tails >80 As being elongated at a rate of 1 nt sec^−1^. Longer time points revealed continued slow extension far beyond 100 As (Fig. 1D). We speculate that this slow mode of poly(A) tail elongation was due to the increased distance between the RNA 3′ end and the CPF-bound Pap1, which is tethered to the PAS sequence (see schematics in Fig. 1C). Here the increasing length of the poly(A) tail reduces the encounters between the RNA 3′ end and the active site of Pap1.

Additionally, a subset of RNAs showed very slow extension (∼30 As in 4 min; ∼0.1 nt sec^−1^) (Fig. 1D). These products became more prominent when *CYC1*_*42*_ was in excess over CPF and were elongated faster at high CPF concentrations (Supplemental Fig. S1A). This suggested distributive polyadenylation by CPF not bound to the PAS sequence in *cis*. This effect appeared to be an artifact of using precleaved RNA substrate, as it was not observed in coupled cleavage and polyadenylation reactions (Supplemental Fig. S1B).

Only ∼20% of the *CYC1*_*42*_ RNA was processively polyadenylated within the first 15 sec, and it took several minutes for the reaction to consume all RNA substrate (Fig. 1D,E), suggesting a rate-limiting step prior to processive elongation. Although this could reflect assembly of an elongation-competent CPAC, the resistance of a fraction of the *CYC1*_*42*_ substrate to both processive and distributive polyadenylation and its delayed polyadenylation at a later time point (e.g. Supplemental Fig. S1A, cf. lanes 4 and 5) hints at an internal activation step. Supporting this, the coupled cleavage and polyadenylation reactions also showed delayed start of polyadenylation, as shown by the accumulation of 5′ cleavage products prior to their subsequent polyadenylation (Supplemental Fig. S1B,C).

In conclusion, CPAC-mediated polyadenylation in vitro can be described by three kinetic reaction phases: (1) a rate-limiting activation step, (2) rapid elongation at 24 nt sec^−1^ up to ∼70 As, and (3) slowed elongation at ∼1 nt sec^−1^ beyond >80 As. We note that the slow elongation of longer tails aligns with the previously reported “intrinsic” poly(A) tail length control, whereby the CPAC can restrict excessive polyadenylation in the absence of PABP regulation (Turtola et al. 2021).

### Poly(A) tail lengths are dictated by a kinetic competition between CPAC-mediated poly(A) tail elongation and Nab2 binding

**Figure 2.**
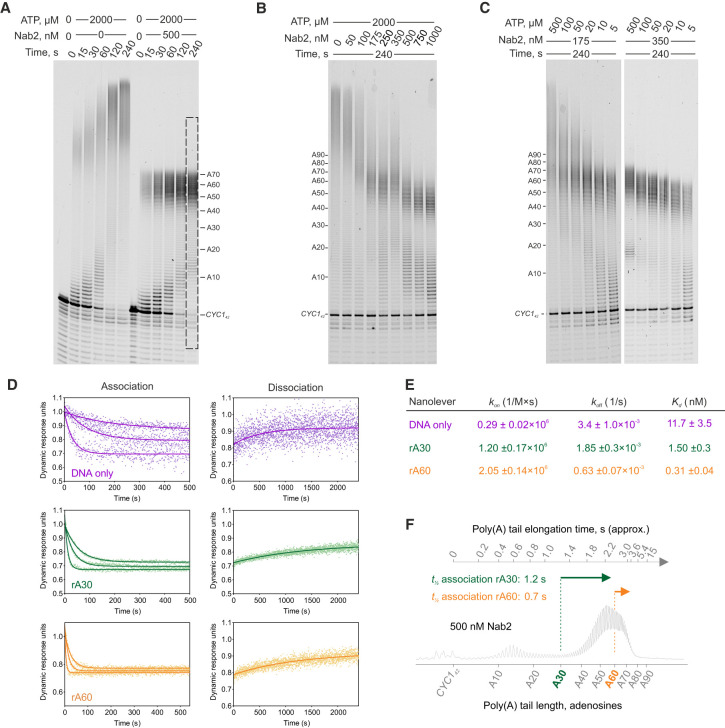
Poly(A) tail lengths are determined by a kinetic competition between CPAC-mediated poly(A) tail elongation and Nab2-mediated termination of poly(A) tail synthesis. (*A*–*C*) The effect of Nab2 on CPAC-mediated *CYC1*_*42*_ polyadenylation analyzed at different time points (*A*), with varying concentrations of Nab2 (*B*) and varying concentrations of ATP (*C*). *CYC1*_*42*_ (25 nM), 75 nM CPF, and 500 nM CF IA/IB were used in all reactions. Gel lane intensity scan of the marked area at 500 nM Nab2 is shown in *F*. (*D*,*E*) SwitchSENSE analysis of Nab2 binding kinetics to poly(A) RNAs tethered to DNA nanolevers. Nab2 (100, 33.3, and 11.1 nM) was flowed over chip-bound nanolevers, and changes in switching speed were used to determine the association rate (*k*_on_). Dissociation of the complex was observed by flowing buffer over the chip surface, resulting in an increase in the DNA switching speed and yielding the dissociation rate (*k*_off_). Curves for DNA nanolever only (magenta), rA_30_ (green), and rA_60_ (orange) were fitted to calculate *k*_on_, *k*_off_, and *K*_*d*_ values and their corresponding standard errors (see the table in *E*). (*F*) Poly(A) tail profile from the gel in *A* plotted against estimated elongation times (from Fig. 1) and Nab2 association half-times (*t*_1/2_) to rA30 (green arrow) and rA60 (orange arrow) at 500 nM Nab2.

To investigate how CPAC-mediated poly(A) tail synthesis is operated in the presence of a PABP, purified Nab2 (Supplemental Fig. S2) was included in the reactions. In the presence of 500 nM Nab2, poly(A) tails were restricted to 50–70 As all through the 4 min incubation (Fig. 2A). Interestingly, increasing Nab2 concentrations led to progressively shorter tails (Fig. 2B), implying that Nab2 binding kinetics play a role in length determination.

To test whether Nab2-mediated termination depends on poly(A) tail elongation rate, we varied ATP concentrations. At 175 nM Nab2, polyadenylation was unrestricted at 500 µM ATP but effectively terminated at lower ATP levels (Fig. 2C, left panel). Likewise, at 350 nM Nab2, the modal poly(A) tail length decreased from ∼60 to ∼45 As as ATP decreased from 500 to 5 µM (Fig. 2C, right panel). Because the same concentration of Nab2 could give rise to different poly(A) tail lengths, this excluded control mechanisms that determine the poly(A) chain length by counting the number of nucleotides. Instead, this suggests that tail length is kinetically determined by competition between the rates of poly(A) tail elongation and Nab2 RNA binding.

To evaluate whether Nab2 association dynamics were compatible with this model, we used SwitchSENSE to determine the association and dissociation rates of Nab2 binding to poly(A) RNAs tethered to DNA nanolevers. Nab2 bound rA_60_ (*k*_on_ = 2.05 × 10^6^ M^−1^ sec^−1^ ± 0.14 × 10^6^ M^−1^ sec^−1^) nearly twice as fast as rA_30_ (*k*_on_ = 1.2 × 10^6^ M^−1^ sec^−1^ ± 0.17 × 10^6^ M^−1^ sec^−1^) and seven times faster than to the DNA nanolever only (Fig. 2D,E). At 500 nM Nab2, the half-times (*t*_1/2_) of Nab2 association to rA_30_ and rA_60_ RNAs were ∼1.2 and ∼0.7 sec, respectively. This aligns well with the 2–3 sec time frame that the CPAC requires to synthesize 50–70 A tails (see Fig. 1C), which are produced in the presence of 500 nM Nab2 (Fig. 2F).

We conclude that Nab2 does not operate as a molecular ruler to measure poly(A) tail length. Rather, the rates of poly(A) tail elongation and Nab2 RNA binding govern a zone of termination, resulting in a restricted distribution of tail lengths. The slowdown of tail elongation after its first ∼70 As efficiently reduces the likelihood of producing hyperadenylated poly(A) tails.

### Poly(A) tail length-dependent dimerization of Nab2

**Figure 3.**
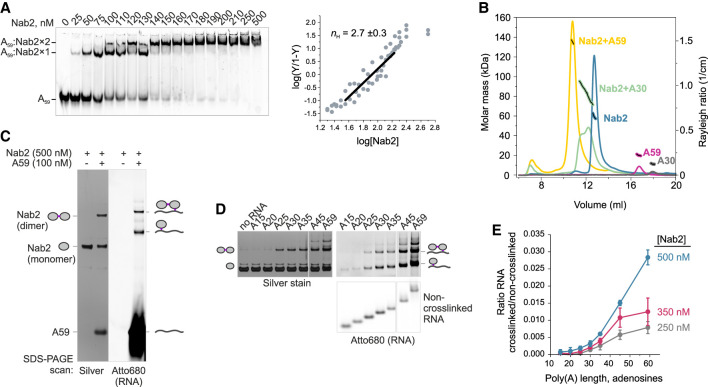
Poly(A) tail length-dependent dimerization of Nab2. (*A*, *left*) EMSA of Nab2 binding to Atto680-labeled A_59_ RNA. (*Right*) Fractional saturation (*Y*) of Nab2 binding to A_59_ was quantified from three experiments and fitted to the Hill equation to determine the Hill coefficient (*n*_*H*_ ± SE). (*B*) Size exclusion chromatography coupled to multiangle light scattering (SEC-MALS) analysis of A_59_ (magenta), A_30_ (gray), Nab2 (blue), and Nab2 complexed with A_59_ (yellow) or A_30_ (green). Light scattering (continuous lines; *Y*-axis at the *right*) and calculated molecular weights across elution peaks (discontinuous black lines with colored outlines; *Y*-axis at the *left*) are shown (see Supplemental Table S1). (*C*) Formaldehyde cross-linking of Nab2 and Atto680-A_59_. Samples treated with 0.3% formaldehyde were separated by denaturing SDS-PAGE, scanned for Atto680 signal (*right* panel) and then silver-stained (*left* panel). Note that the Nab2–RNA cross-links are formed with lower efficiency than Nab2–Nab2 protein cross-links and remain below the detection limit of silver staining. (*D*) Cross-linking of 500 nM Nab2 with 100 nM Atto680-labeled poly(A) RNAs of various lengths. The gel panels were cropped from the same gel. (*E*) Quantification of Nab2–RNA cross-links at 500, 350, or 250 nM Nab2. Corresponding scans and silver-stained gels are shown in Supplemental Figure S3B.

Dissociation constants (*K*_*d*_) derived from the Nab2 on and off rates indicated approximately fivefold higher affinity for A_60_ RNA than A_30_ (Fig. 2E), prompting further biochemical analysis of Nab2:poly(A) RNPs. Electrophoretic mobility shift assays (EMSAs) using fluorescently labeled A_59_ RNA revealed multiple shifted bands, indicating binding of one or more Nab2 molecules (Fig. 3A, left). The positive Hill coefficient (*n*_*H*_ = 2.7) suggested that cooperative interactions between monomeric units of Nab2 enhance its RNA binding (Fig. 3A, right).

To determine the Nab2 oligomerization state more precisely, we used size exclusion chromatography coupled to multiangle light scattering (SEC-MALS). Nab2 (*M*_*r*_; 59.1 kDa) and A_59_ RNA (*M*_*r*_; 19.4 kDa) formed a 135 kDa complex, consistent with a 2:1 Nab2:RNA stoichiometry, which was further supported by the conjugate analysis that separated the total molecular weight to protein and RNA components (Fig. 3B; Supplemental Table S1). Doubling the ratio of Nab2 to RNA in the sample did not yield higher-order complexes. Separately, Nab2 or A_59_ each eluted as a monomer, demonstrating that Nab2 dimerization is RNA-dependent. Interestingly, a complex formed between Nab2 and the A_30_ RNA (*M*_*r*_; 9.9 kDa) migrated as a bimodal peak between the dimeric and monomeric Nab2 species. Light scattering and UV signals (Supplemental Fig. S3A) suggested that one Nab2 molecule bound one to three molecules of A_30_ RNAs (Supplemental Table S1), indicating that the stability of the Nab2 dimer is sensitive to poly(A) RNA length.

To quantify this length dependence, we used formaldehyde cross-linking to capture the Nab2–poly(A) RNA interaction. As shown by silver staining in Figure 3C, intermolecular cross-links between two molecules of Nab2 were observed in the presence of the A_59_ RNA but not in its absence. Cross-links between Nab2 and A_59_ were also detected but formed with lower efficiencies. Because the efficiency of RNA–protein cross-linking was sensitive to the concentration of Nab2 (Supplemental Fig. S3B), we used it semiquantitatively to assess binding across RNAs of varying lengths. At 500 nM Nab2, A_59_ RNA yielded ∼3% cross-linking in 10 min, whereas A_15_ and A_20_ showed only 0.08% and 0.1%, respectively (Fig. 3D,E). Cross-linking efficiency increased markedly with >25 adenosine RNAs, and Nab2 dimer cross-links followed the same trend. Thus, low cross-linking efficiency of short RNA could not be explained solely by fewer cross-linkable sites. These results therefore suggested that Nab2 dimerization requires continuous poly(A) RNA of at least 25 adenosines and that the interaction between Nab2 and RNA becomes progressively more stable as the poly(A) tail length increases.

### Dimerized Nab2 terminates polyadenylation

**Figure 4.**
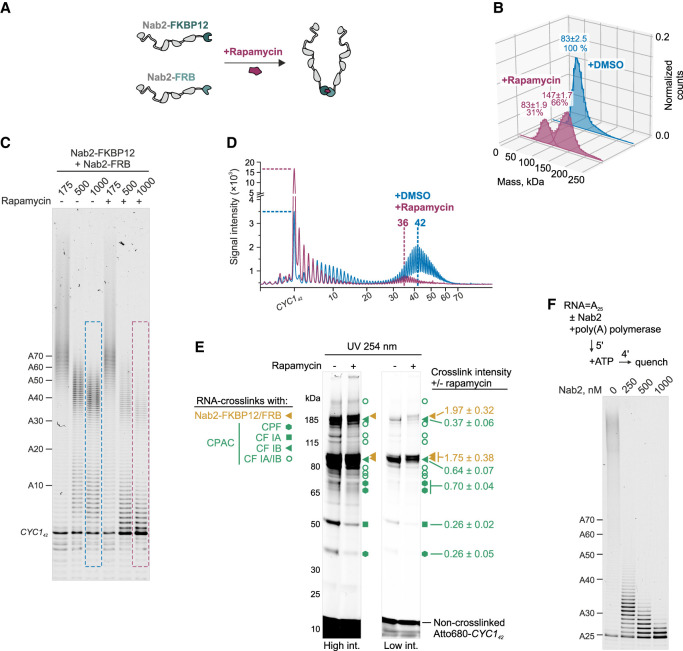
Dimerized Nab2 inhibits polyadenylation. (*A*) The principle of rapamycin-induced dimerization of Nab2-FKBP12 and Nab2-FRB fusion proteins. (*B*) Mass photometric analysis of 125 nM Nab2-FKBP12 and 125 nM Nab2-FRB with 2.5% (v/v) DMSO or 1 µM rapamycin. The Gaussian fits of count-normalized histograms show peak positions ± SEM (in kilodaltons) and the percentage of total counts. (*C*) The effect of rapamycin-induced Nab2 dimerization on *CYC1*_*42*_ polyadenylation by the CPAC. Nab2-FKBP12 and Nab2-FRB (each at half of the total Nab2 concentration indicated) were preincubated with DMSO or 1 µM rapamycin before being added to the polyadenylation reactions with ATP. The reactions were stopped after 4 min. (*D*) Gel lane intensity scans from *C.* Horizontal lines indicate nonadenylated *CYC1*_*42*_ intensities, and vertical lines mark modal poly(A) tail lengths (>25 As). (*E*) SDS-PAGE of UV-induced RNA–protein cross-links detected via Atto680 fluorescence from the RNA. Reactions contained 25 nM *CYC1*_*42*_, 75 nM CPF, 500 nM each CF IA/IB, 500 nM each Nab2-FKBP12/FRB, and either DMSO or 1 µM rapamycin. The intensities of cross-linked RNA–protein adducts assigned to Nab2-FKBP12/FRB and CPAC subunits (gold and green symbols, respectively) (see Supplemental Fig. S4C) were quantified from three replicates (±rapamycin). Mean intensity ratios ± SD are shown beside each band. (*F*) The effect of 0–1000 nM Nab2 on polyadenylation of A_25_ RNA by 3000 nM poly(A) polymerase. Polyadenylation reactions were carried out in Mn^2+^-containing buffer (see the Materials and Methods) to enhance polymerase activity.

To test the functional role of Nab2 dimerization, we used rapamycin-induced heterodimerization of FKBP12 and FRB domains (Choi et al. 1996) to conditionally tether two Nab2 molecules (Fig. 4A). We constructed and purified fusion proteins where FKBP12 or FRB domains were linked to the C terminus of Nab2 (Supplemental Fig. S2). Mass photometry confirmed that Nab2-FKBP12 and Nab2-FRB proteins were monomeric in solution but formed a heterodimeric complex in the presence of rapamycin (Fig. 4B). In contrast, unmodified Nab2 required poly(A) RNA to form a ternary complex (Supplemental Fig. S4A).

Nab2-FKBP12 and Nab2-FRB proteins were capable of terminating CPAC-mediated polyadenylation both independently and in combination (Supplemental Fig. S4B). Thus, the fused domains did not interfere with termination activity. Notably, when rapamycin was added before initiating the reaction, *CYC1*_*42*_ polyadenylation was largely prevented (Fig. 4C). Furthermore, a small fraction of substrate RNA that escaped this early inhibition was polyadenylated to a modal length of ∼36 As at 1 µM Nab2, compared with ∼42 As in a reaction without rapamycin (Fig. 4D). Rapamycin itself did not affect the polyadenylation activity of the CPAC (Supplemental Fig. 4B). These findings suggest that Nab2 can inhibit the CPAC both before and during polyadenylation and that promoting intermolecular Nab2–Nab2 interactions enhances this inhibitory effect.

To investigate how rapamycin-linked Nab2-FKBP12 and Nab2-FRB inhibit polyadenylation initiation, we used 254 nm UV-cross-linking to capture direct RNA–protein contacts before ATP addition and then separated the cross-linked RNA–protein complexes by SDS-PAGE. In the control sample, the *CYC1*_*42*_ RNA cross-linked to several CPAC subunits and weakly to Nab2-FKBP12/FRB (Fig. 4E; Supplemental Fig. S4C). Strikingly, the addition of rapamycin increased Nab2-FKBP12/FRB cross-links and reduced those to the CPAC, indicating that the chemically induced Nab2 dimer prevents initiation by outcompeting the CPAC for binding to the nonadenylated RNA.

The findings shown in Figures 2–4 suggest that wild-type Nab2 dimerizes after the CPAC has synthesized a poly(A) tail longer than ∼25 As, which then terminates polyadenylation. Because the CPAC is already assembled on the RNA at this stage, the data imply another mode of inhibition that impacts poly(A) tail elongation. To define the minimal requirements for this termination activity, we performed reactions using only poly(A) polymerase and A_25_ RNA. Under these simplified conditions, Nab2 still inhibited polyadenylation in a concentration-dependent manner (Fig. 4F), indicating that poly(A)-bound Nab2 dimers can block polymerase access to the RNA 3′ end independently of other CPAC components.

Curiously, the Nab2 concentration required for terminating elongation was similar with or without rapamycin-induced tethering (Fig. 4C). Given the cooperativity of Nab2 binding to poly(A) RNA (Fig. 3A), this supports a model in which two monomers first bind poly(A) RNA independently and then interact to assemble a stable dimer that terminates polyadenylation.

### Nab2 CCCH zinc fingers 5–7 mediate poly(A) length sensing, dimerization, and termination of poly(A) tail synthesis

**Figure 5.**
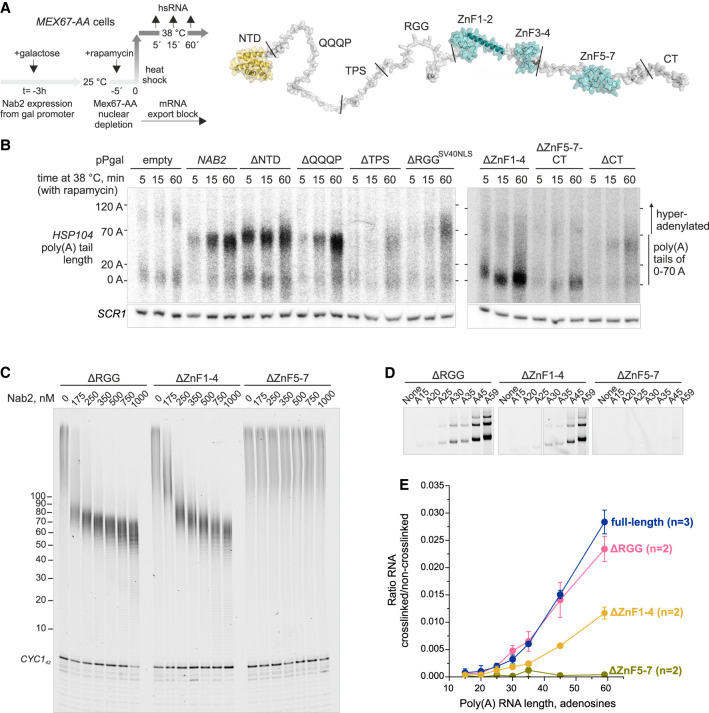
CCCH zinc fingers 5–7 are essential for poly(A) tail length sensing. (*A*) Experimental scheme to measure nuclear mRNA poly(A) tail lengths in *MEX67-AA* cells expressing Nab2 mutants. (*Left*) Nab2 variants were overexpressed for 3 h, after which mRNA export was blocked by adding rapamycin 5 min before a 38°C heat shock. RNA samples were collected at the indicated time points. (*Right*) AlphaFold model of Nab2 (Varadi et al. 2022), stretched for clarity. Structured N-terminal domain (NTD) and ZnFs are shown in yellow and teal, and predicted disordered regions are in gray. (*B*) RNase H/Northern analysis of *HSP104* RNA 3′ ends. Cells depleted of nuclear Mex67 were incubated for varying durations at 38°C while expressing endogenous levels of Nab2 (pPgal empty) or overexpressing full-length (pPgal-*NAB2*) or mutant Nab2 from a pPgal plasmid. See Supplemental Figure S5A for protein levels. *HSP104* RNA was detected using a probe near the PAS sequence. To increase resolution, transcripts were cleaved by RNase H using an antisense oligo 230 nt upstream of the PAS sequence. Migration of normally adenylated (0–70 As) and hyperadenylated (>70 As) transcripts is indicated. *SCR1* RNA served as a loading control. (*C*) CPAC-mediated *CYC1*_*42*_ polyadenylation with Nab2 truncation mutants. See Supplemental Figure S2 for purified proteins. (*D*) Formaldehyde cross-linking between 500 nM Nab2 variants and 100 nM Atto680-labeled poly(A) RNAs. Panels were cropped from two gels run and exposed in parallel. See Supplemental Figure S5C for full RNA gel scans and silver-stained images. (*E*) Quantification of RNA–Nab2 cross-links from *D*. The full-length Nab2 data (blue) are reproduced from Figure 3E.

To dissect the molecular basis for poly(A) tail termination activity, we next characterized Nab2 mutant variants using in vivo and in vitro approaches. Poly(A) tail length control was examined in vivo using our previously established assay that decouples nuclear polyadenylation from cytoplasmic deadenylation by blocking mRNA export in the Mex67 anchor away (*MEX67-AA*) strain (Fig. 5A, left). Export block sequesters endogenous Nab2 on nuclear-restricted poly(A) tails, preventing Nab2-mediated poly(A) tail length control. However, overexpression of exogenous Nab2 restores length control (Turtola et al. 2021). Accordingly, detection of poly(A) tail lengths using RNase H digestion and Northern blotting showed that the heat-inducible *HSP104* mRNA tails were hyperadenylated (>70 As) in export-blocked cells but restricted to ∼60 As with Nab2 overexpression (Fig. 5B, cf. pPgal “empty” and “*NAB2*”). Nab2 also stabilizes nascent RNAs by preventing nuclear degradation (Schmid et al. 2015; Tudek et al. 2018), as reflected by higher *HSP104* mRNA levels in Nab2-overexpressing cells.

Nab2 consists of an N-terminal domain (NTD), seven CCCH-type ZnFs (with ZnF1–2 and ZnF3–4 folding into tandem ZnF domains and ZnF5–7 forming an ordered domain), and disordered regions including the glutamine–proline-rich region (QQQP), the sequence surrounding the Slt2 phosphosite (TPS), an arginine–glycine–glycine-rich region (RGG), and the C-terminal tail (CT) (Truant et al. 1998; Marfatia et al. 2003; Grant et al. 2008; Carmody et al. 2010; Brockmann et al. 2012; Martínez-Lumbreras et al. 2013) (Fig. 5A, right). To reveal regions essential for tail length control, we tested Nab2 truncations. Cells expressing ΔNTD, ΔQQQP, ΔTPS, or ΔCT Nab2 variants restricted tail length similar to full-length Nab2 (Fig. 5B; Supplemental Fig. S5A). In contrast, poly(A) tail lengths were not restricted in cells that expressed the ΔRGG^SV40NLS^ (SV40 nuclear localization signal [NLS] inserted at the N terminus to compensate for the lack of NLS in the ΔRGG protein), ΔZnF1–4, or ΔZnF5–7-CT Nab2 variants.

To characterize their function in vitro, we purified the ΔRGG, ΔZnF1–4, and ΔZnF5–7 Nab2 proteins (Supplemental Fig. S2). Although ΔRGG and ΔZnF1–4 required higher concentrations and allowed longer tails, they still terminated polyadenylation (Fig. 5C). In contrast, the ΔZnF5–7 failed to terminate polyadenylation at any tested concentration. Furthermore, this protein did not form formaldehyde-mediated RNA–protein or Nab2–Nab2 cross-links (Fig. 5D; Supplemental Fig. S5C). Cross-linking by ΔRGG resembled full-length Nab2, whereas ΔZnF1–4 displayed reduced but still tail length-dependent cross-linking, with a cutoff at 25 As (Fig. 5E).

We conclude that although ZnF1–4 and RGG promote the association of Nab2 with the poly(A) tail during polyadenylation, the ZnF5–7 domain senses the minimal poly(A) tail length leading to Nab2 dimerization, in turn terminating poly(A) tail synthesis.

### Cooperation of CCCH zinc fingers is required for Nab2 functions

A key role of ZnF5–7 in poly(A) RNA binding and poly(A) tail length control aligns with earlier studies (Viphakone et al. 2008; Kelly et al. 2010; Brockmann et al. 2012). Aibara et al. (2017) showed that isolated ZnF5–7 homodimerizes on A_11_G RNA, forming two poly(A) binding sites on opposite sides of the dimer. We hypothesized that this structure might explain the observed 25 adenosine minimum for Nab2 binding, as the RNA chain must be long enough to bridge both sites (Supplemental Fig. S6A).

**Figure 6.**
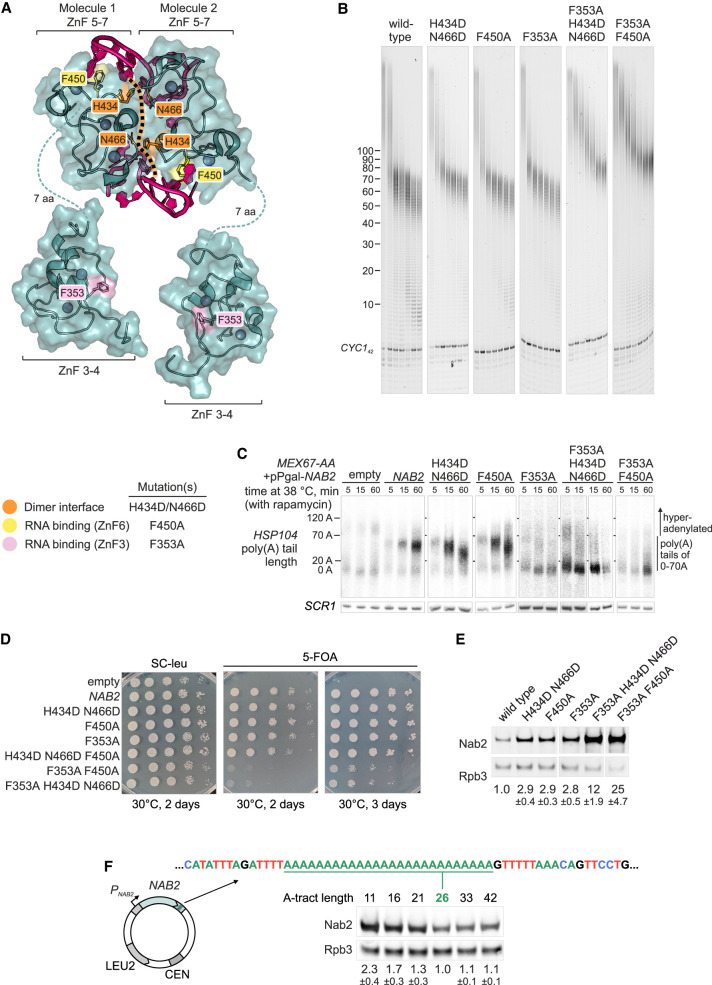
Nab2 function is tuned by the cooperation between ZnFs and the autoregulatory control of protein levels. (*A*) Composite model of the Nab2 ZnF5–7:A_11_G heterotetramer (PDB 5L2L) (Aibara et al. 2017) with ZnF3–4 modules (PDB 3ZJ2) (Martínez-Lumbreras et al. 2013). The dimer interface (dotted line) includes H434 and N466 (orange). RNA-binding residues F450 and F353 are in yellow and pink, respectively. (*B*) CPAC-mediated *CYC1*_*42*_ polyadenylation reactions with wild-type and mutant Nab2 proteins at 0, 175, 250, 350, 500, 750, and 1000 nM. See Supplemental Figure S2 for proteins and Supplemental Figure S6B for the side-by-side comparison of 1000 nM Nab2 samples. (*C*) Nuclear *HSP104* mRNA poly(A) tail lengths in cells expressing Nab2 mutants using the assay presented in Figure 5, A and B; see expression levels in Supplemental Figure S6C. (*D*) Spot dilution growth assays of the Δ*nab2::HIS3* p(*NAB2/CEN, URA3*) strain transformed with p(*NAB2*/*CEN, LEU2*) plasmids expressing wild-type, mutant, or no Nab2 (empty) under the native promoter and downstream sequences. See Supplemental Figure S7A for growth at 25°C and 37°C. (*E*,*F*) Western blot analysis of Nab2 and Rpb3 protein levels in strains expressing Nab2 point mutants (*E*) or A tract variants (*F*). Nab2/Rpb3 ratios (mean ± SD; two to five replicates) normalized to wild type are shown *below*. The plasmid structure in *F* indicates the autoregulatory downstream sequence for the wild-type A tract (A26^WT^; underlined). Panels in *E* are cropped from the same gel.

To test this, we generated a dimer interface mutant (H434D/N466D) introducing electrostatic repulsion without directly affecting Nab2:poly(A) RNA interactions and a F450A mutant (Brockmann et al. 2012) directly disrupting RNA binding to ZnF6 (Fig. 6A). Both mutant proteins retained termination activity but yielded slightly longer poly(A) tails than wild-type Nab2 in vitro (Fig. 6B; Supplemental Fig. S6B) and in vivo when observed after 5 min of *HSP104* induction (Fig. 6C). However, the most notable effect in vivo was the shortening of poly(A) tails of the nuclear-restricted *HSP104* mRNAs within 15–60 min after heat shock. These effects became even more visible when the dimer interface was further destabilized by additional substitutions (Supplemental Fig. S6A,C,D). Formaldehyde cross-linking indicated that both sets of mutations reduced the binding of Nab2 to poly(A) RNA (Supplemental Fig. S6E,F). The RNA–protein cross-linking profile of the dimer interface mutant showed a reduced amount of the dimeric form compared with wild-type Nab2 (Supplemental Fig. S6G). Thus, intermolecular protein contacts stabilize the Nab2:poly(A) RNP.

The mild tail length phenotypes of these mutants led us to consider regions outside of ZnF5–7. Based on the results from Figure 5, we further examined individual ZnF1–4 and the connecting linkers between ZnF2 and ZnF3 (L2–3) and between ZnF4 and ZnF5 (L4–5). Deletion analysis indicated that ZnF3 and ZnF4, which fold into one module (Martínez-Lumbreras et al. 2013), were essential for termination, whereas ZnF1, ZnF2, and the linkers were dispensable (Supplemental Fig. S5A,B). Mutating the ZnF3 residue F353, which was previously implicated in poly(A) RNA binding (Martínez-Lumbreras et al. 2013), compromised poly(A) tail length control both in vitro and in vivo (Fig. 6B,C; Supplemental Fig. S6B–D), demonstrating the need for ZnF3 for timely termination of polyadenylation.

Notably, combining the ZnF3 mutation F353A with the dimer interface mutant or the ZnF6 mutation F450 led to significantly longer poly(A) tails, and higher concentrations of proteins were required for termination in vitro, with consistent pronounced defects observed in vivo (Fig. 6B,C). Similar results were obtained by using alternative ZnF3 RNA-binding mutants (Supplemental Fig. S6A,C,D; Martínez-Lumbreras et al. 2013).

Collectively, these results demonstrate that proper function of Nab2 requires the cooperation between different ZnF modules, each providing interactions that stabilize poly(A) RNA binding either directly by contacting RNA or indirectly by stabilizing the protein–protein interface of the dimer.

### Autoregulation of *NAB2* calibrates Nab2 protein levels in a poly(A) RNA binding-dependent manner

Having established the functional importance of the intramolecular and intermolecular cooperation of Nab2 for its functions, we next assessed how impairing these different RNA-binding modes impacts cell viability. To this end, wild-type or point mutant *NAB2* alleles were expressed from single-copy *LEU2*-based plasmids under the control of a native promoter and 3′ UTR. These plasmids were introduced into a Δ*nab2* strain maintained by a *URA3*-based *NAB2* plasmid, which could be counterselected on media containing 5-FOA. In line with the *NAB2* gene being essential (Hector et al. 2002; Marfatia et al. 2003), cells carrying an empty *LEU2* plasmid failed to grow on 5-FOA (Fig. 6D; Supplemental Fig. S7A). Separate mutations targeting the dimer interface or RNA binding supported growth comparable with that of wild-type *NAB2*. In contrast, strains expressing multisite mutations in both ZnF3 and ZnF5–7 grew noticeably slower at 25°C, 30°C, and 37°C.

Given Nab2's concentration-dependent inhibition of polyadenylation (Fig. 2) and the increased concentrations required for mutant proteins (Fig. 6B), we estimated Nab2 levels by Western blot. Strikingly, all mutant proteins accumulated to higher levels than wild type (Fig. 6E). ZnF3 and ZnF5–7 mutants showed threefold to fourfold increases, whereas multisite mutants accumulated 12-fold to 25-fold more protein (normalized to Rpb3) (Fig. 6E; Supplemental Fig. S7B).

These effects could be reconciled with previously reported Nab2 autoregulation, which involves Nab2 binding to a genomically encoded A_26_ tract in the *NAB2* pre-mRNA to inhibit its own expression (see the Discussion; Roth et al. 2005, 2009; Ghazal et al. 2009). Shortening this A tract to A_21_, A_16_, or A_11_ increased wild-type Nab2 protein expression 1.3-fold, 1.7-fold, or 2.3-fold, respectively (Fig. 6F). In contrast, lengthening it to A_33_ or A_42_ had little effect. Therefore, reduced binding of the wild-type Nab2 to the autoregulatory A tract mimics the effects of mutant Nab2 proteins with impaired poly(A) RNA binding. Increased cellular concentrations of Nab2 mutants supposedly compensate for their reduced poly(A) binding activity, thereby restoring function completely (as in the case of the separate ZnF mutants) or partially (as in the case of the multisite mutants). We conclude that *NAB2* autoregulation calibrates the poly(A) binding capacity of Nab2, thereby providing a robust control of poly(A) tail synthesis and mRNA stability in the nucleus.

## Discussion

### A kinetic ruler controls nuclear poly(A) tail length

By tracking RNA polyadenylation reactions with high time resolution and linking the biochemical properties of Nab2:poly(A) RNPs to reaction termination kinetics, we uncovered the molecular basis of mRNA poly(A) tail length control in *S. cerevisiae*. Termination of polyadenylation by Nab2 requires its dimerization on the nascent poly(A) RNA, which can occur once the poly(A) tail is at least 25 As long. This explains how Nab2 avoids terminating polyadenylation prematurely. The poly(A):Nab2 RNP likely forms as the RNA chain wraps around a Zn5–7 dimer, which might induce a conformational change or steric block to inhibit polyadenylation. The minimal length for Nab2 action is consistent with a nondiscrete nuclease-protected footprint of poly(A) RNA-bound Nab2 that peaks at ∼23–25 nt (Viphakone et al. 2008) and with the ZnF5–7:A_11_G crystal structure (Aibara et al. 2017). The high cooperativity of binding is explained by ZnF–RNA interactions that are maximized within the dimeric ZnF5–7 unit and stabilized by the protein–protein contacts at the dimer interface. Additional contacts via tandem ZnFs and the RGG domain may promote efficient termination by increasing the association rates and the RNA residence times prior to dimerization.

**Figure 7.**
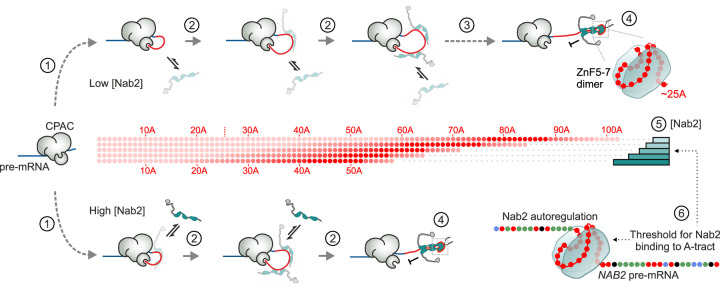
Kinetic ruler mechanism of poly(A) tail length control. Polyadenylation of pre-mRNA (blue) is shown in the presence of low (*top*) or high (*bottom*) Nab2 concentrations (gray/teal). (1) The CPAC (*gray*) binds the PAS sequence and initiates the synthesis of a poly(A) tail (red) by tethering poly(A) polymerase. Elongation begins after a slow initiation step (curved dashed arrows). (2) The first 70 As are added rapidly (∼3 sec; short gray arrows). The Nab2 binding rate (black arrows) increases as the tail grows. (3) CPAC activity slows after ∼70 As (long dashed arrow). (4) Nab2 dimerizes on tails >25 As via ZnF5–7 and stabilized by other ZnFs, terminating polyadenylation. (5) Nab2 concentration determines poly(A) tail length: Low Nab2 (light teal bars) allows longer tails, and high Nab2 (dark teal bars) results in shorter tails. (6) Nab2 levels are autoregulated through its binding to an A_26_ sequence within the *NAB2* pre-mRNA. The binding properties of Nab2 to this sequence lock its nuclear-available concentration.

Importantly, neither the minimal RNA footprint nor the binding architecture of Nab2 alone is sufficient to explain how the protein restricts poly(A) tail lengths to ∼60 As. We solve this conundrum by considering polyadenylation kinetics, demonstrating that poly(A) tail length control is a nonequilibrium process: Tail length is set by the relative rates of CPAC-mediated polyadenylation and Nab2–poly(A) association. As a result, poly(A) tail length is not “measured” per se; rather, the on rate of Nab2 binding to the poly(A) tail decides its length. The on rate increases with increasing tail length and is proportional to the concentration of Nab2. We therefore propose that Nab2 is a “kinetic ruler,” the concentration of which is used for quantifying poly(A) tail length (Fig. 7).

### Calibration and tunability of the kinetic ruler

Although the suggested kinetic ruler provides a solution to the problem of RNA chain length sensing, such a fine-tuned mechanism requires careful titration of Nab2 protein concentrations in order to maintain the production of mRNAs with specific poly(A) tail lengths. This is ingeniously managed by an autoregulatory system in which Nab2 negatively impacts the production of its own mRNA. Here, binding of Nab2 to an encoded A_26_ tract within the *NAB2* pre-mRNA prevents the cotranscriptional utilization of the PAS sequence by the CPAC. This leads to an alternative RNase III-triggered transcription termination event, which in turn elicits exosome-mediated degradation of the *NAB2* pre-mRNA. Conversely, if Nab2 is not available, the CPAC cleaves the pre-mRNA upstream of the A_26_ sequence and produces a mature *NAB2* mRNA (Roth et al. 2005, 2009; Ghazal et al. 2009).

According to the principles of autoregulatory gene networks (Alon 2007), the binding affinity of Nab2 to the A_26_ tract determines and locks the steady-state level of nuclear-available Nab2. Consistently, we observed drastic upregulation of the levels of mutant Nab2 proteins with reduced ability to bind poly(A) RNA. Our results further suggest that the A_26_ tract promotes Nab2 dimerization (Fig. 3), which in turn could block CPAC assembly on a nearby PAS sequence (Fig. 4). These A tracts, also found downstream from various *NAB2* homologs in other fungi (e.g., 51 As in a 57 nt stretch in *Candida auris*), may have evolved to calibrate the dimer-forming capacity of Nab2 and tune global mRNA poly(A) tail lengths (Fig. 7).

Nab2 availability might still fluctuate on timescales shorter than the response time to establish a new equilibrium or display a nonuniform distribution within the nucleus, with consequences for mRNA production. For example, Nab2 becomes sequestered into foci following nuclear accumulation of poly(A) RNA in conditions such as heat shock (Carmody et al. 2010) and glucose depletion (Heinrich et al. 2024) or as a result of defects in several RNA processing pathways that impair mRNA export (Paul and Montpetit 2016; Tudek et al. 2018; Aguilar et al. 2020). The kinetic ruler model now rationalizes the hyperadenylation phenotype observed in yeast strains impaired in mRNP processing and export (Hilleren and Parker 2001; Jensen et al. 2001; Libri et al. 2002; Rougemaille et al. 2007) and predicts that mRNA poly(A) tail lengths respond dynamically to Nab2 availability. Nab2 regions such as QQQP, TPS, and RGG influence its biomolecular clustering properties, nuclear–cytoplasmic shuttling behavior, and interactions with mRNP proteins (Marfatia et al. 2003; Iglesias et al. 2010; Heinrich et al. 2024) and may be subjected to regulation via post-translational modifications (Green et al. 2002; Carmody et al. 2010; Insco et al. 2023). An intriguing question for future studies is therefore whether Nab2 modifications may recalibrate the kinetic ruler to alter poly(A) tail lengths, thereby tuning regulatory properties of individual RNAs or across the entire transcriptome.

### Distinct poly(A) tail elongation and termination mechanisms in yeast and mammals

Despite the high level of conservation of the CPAC machineries across eukaryotes, poly(A) tails are elongated in distinctly different manners in yeast and mammalian cells. A major difference is the basal polyadenylation processivities: Yeast Pap1 is tightly bound to CPAC and synthesizes poly(A) tails efficiently up to ∼70 As (this study), whereas the mammalian PAP, which is more loosely associated with the CPAC machinery, requires additional stimulation by PABPN1 during poly(A) tail elongation (Kerwitz et al. 2003; Eckmann et al. 2011; Rodríguez-Molina and Turtola 2023). In addition, the kinetic ruler model of tail length control in yeast fundamentally differs from the proposed model in mammals. In the current view, mammalian mRNA poly(A) tail length is determined by a molecular ruler mechanism whereby ∼15–20 molecules of PABPN1 cover the ∼250 A poly(A) tail and terminates the processive phase of tail elongation by obstructing interactions between PAP, PABPN1, and the rest of the CPAC machinery (Keller et al. 2000; Kühn et al. 2009). However, this picture of mammalian polyadenylation control is confounded by observations from several organisms, indicating that the depletion of Nab2/ZC3H14 leads to increased lengths of bulk poly(A) tails (Pak et al. 2011; Kelly et al. 2014; Bienkowski et al. 2017; Rha et al. 2017; Morris and Corbett 2018; Li et al. 2024). This raises the possibility that Nab2/ZC3H14 may regulate the timing of poly(A) tail termination alongside PABPN1. Notably, the human ZC3H14 and yeast Nab2 appear to share the RNA binding modality needed for termination, as overexpression of a chimeric protein (where ZnF1–7 of yeast Nab2 were replaced by ZnF1–5 of human ZC3H14) controlled poly(A) tail length and protected the nuclear-restricted *HSP104* mRNA in yeast (Supplemental Fig. S5A,D). Further studies are needed to clarify the roles and possible interplay of different factors in this process.

### Architectural and kinetic considerations of PABP-mediated regulation of RNA processing

Poly(A) tail-bound nuclear PABPs form protective structures at RNA 3′ ends and promote mRNA export yet can also act within different adaptor complexes to target various transcript classes for decay, but the logic behind these choices remains largely unknown (for review, see Rambout and Maquat 2024). Nab2/ZC3H14 has been mostly linked to protective functions (Grenier St-Sauveur et al. 2013; Schmid et al. 2015; Morris and Corbett 2018; Tudek et al. 2018; Turtola et al. 2021; Latour et al. 2025), whereas PABPN1 works in several decay pathways (Bresson et al. 2015; Meola et al. 2016; Latour et al. 2025). The characterization of Nab2 in this study suggests profound differences in the biochemical properties of ZC3H14 compared with those of PABPN1 that might provide clues to their antagonistic functions.

First, our mutagenesis revealed the requirement of Nab2 dimerization and multidomain interactions in protecting the 3′ ends of long poly(A) tails against polymerase and nuclease activities, thus showing how it preserves poly(A) tail lengths in the nucleus. In contrast, PABPN1 leaves the RNA 3′ ends accessible for polymerase and nuclease activities (Kerwitz et al. 2003; Nguyen et al. 2015). Second, Nab2 proteins bind poly(A) RNA in a cooperative manner, conferring a preference to bind >25 A tails. In contract, PABPN1binds 11 As without notable cooperativity emerging for longer poly(A) RNA substrates (Meyer et al. 2002). Therefore, poly(A) tail length-dependent recruitment of different PABPs might be one mechanism to distinguish between transcript types and direct them to different fates.

Echoing the tunable kinetic ruler mechanism proposed here, the coupling of poly(A) tail length to translational efficiency in oocytes and early embryos requires a concentration regime of PABPC that favors its association with long-tailed mRNAs (Xiang and Bartel 2021). Intriguingly, Pab1 (the yeast homolog of PABPC) can also inhibit polyadenylation and control poly(A) tail lengths in a concentration-dependent manner (Viphakone et al. 2008; Turtola et al. 2021), raising the possibility that the length control principles uncovered here could play a role in the complex dynamics of cytoplasmic polyadenylation and deadenylation (Lim et al. 2016; Xiang et al. 2024). Our results therefore highlight the need to understand both structural and kinetic properties behind PABP-mediated regulatory mechanisms.

## Materials and methods

### Protein expression and purification

CPF modules (polymerase with Pfs2-SII and nuclease-phosphatase with Ref2-SII), CF IA (with SII-Pcf11 and 8xHis-Rna14), and SII-Nab2 were expressed in insect cells with the baculovirus expression system (Hill et al. 2019; Kumar et al. 2021; Turtola et al. 2021). CF IB (6xHis-Hrp1) (Casañal et al. 2017) and Nab2-6xHis were expressed in Xjb RIL *Escherichia coli* cells. Plasmids are listed in Supplemental Table S2. Full protocols for expression and purification are described in the Supplemental Material. CPF was reconstituted from a purified polymerase module and the nuclease-phosphatase modules as described previously by Rodríguez-Molina et al. (2022).

### Polyadenylation reactions

Reactions with CPAC (modified from Turtola et al. 2021) were performed at 30°C in polyadenylation buffer [25 mM HEPES-KOH at pH 8.0, 150 mM KOAc, 2 mM Mg(OAc)_2_, 0.05 mM EDTA, 0.1 U/µL RiboLock (Thermo Scientific)]. Most reactions were set up by preincubating the RNA with CF IA/IB (in one-third of the final reaction volume) for 5 min, and then 0.33 vol of CPF ± Nab2-6xHis was added and preincubated for an additional 3 min, followed by 0.33 vol of ATP. See Supplemental Table S4 for oligo sequences and Supplemental Table S5 for the specification of conditions and final concentrations. Reactions involving only yeast poly(A) polymerase (final concentration 35 U/µL; Jena Bioscience RNT-006) were performed in poly(A) polymerase reaction buffer (20 mM Tris-HCl at pH 7.0, 0.6 mM MnCl_2_, 0.02 mM EDTA, 0.02 mM DTT, 0.1 mg/mL acetylated BSA, 10% [v/v] glycerol, 0.1 U/µL RiboLock, 0.5 mM ATP). Reactions were stopped after 4 min (or at the indicated times) with 0.7 vol of 2 M HCl and neutralized with 5.3 vol of neutralization solution (290 mM Tris base, 13 mM EDTA, 0.025% [w/v] bromophenol blue, 94% formamide). Time-resolved polyadenylation reactions used an RQF 3 quench ﬂow (KinTek Corporation). CPAC (16 µL) was mixed with 16 µL of 4 mM ATP, quenched with 83 µL of 0.5 M HCl, and neutralized with 165 µL of neutralization solution. RNAs were separated on 13% denaturing polyacrylamide gels and visualized with Odyssey (Li-Cor Biosciences) or Sapphire (Azure Biosystems) imagers. Gel scans were quantiﬁed with ImageJ. Poly(A) tail length at each time point was quantified in Origin by subtracting background from gel lane intensity profiles and integrating the signal across the poly(A) wave front. The midpoint of the cumulative signal was used to estimate the mean poly(A) tail length of the elongating RNA population. Linear fitting was performed with the instrumental weighing method.

### SwitchSENSE

SII-Nab2 binding to RNA and DNA was analyzed on a DRX2 instrument (Dynamic Biosensors) using an MPC2-48-2-G1R1-S chip at 25°C in SwitchSENSE buffer [12.5 mM HEPES-KOH at pH 8.0, 75 mM KOAc, 1 mM Mg(OAc)_2_, 0.025 mM EDTA]. Prior to each kinetic analysis, oligonucleotides cNLA-X2 with CX2 and cNLB-X1 with either CX1-rA30 or CX1-rA60 (Supplemental Table S4) together were annealed to DNA strands attached on the chip surface (NL-A48 and NL-B48, respectively) by flowing 200 nM oligos over the chip for 4 min in 10 mM Tris-HCl (pH 7.4), 40 mM NaCl, 0.05% (v/v) Tween 20, 50 µM EDTA, and 50 µM EGTA. As a result, 68 bp dsDNA nanolevers were formed without (DNA) or with a 30 nt (rA30) or 60 nt (rA60) RNA overhang extending in the 5′-to-3′ direction. SII-Nab2 was injected (1:3 dilution from 100 nM) at 50 µL/min for 9 min followed by dissociation in SwitchSENSE buffer after the highest concentration for 80 min. Data were fitted with SwitchBUILD software using a 1:1 kinetic model to obtain the rate constants for association (*k*_on_) and dissociation (*k*_off_) and to calculate the kinetic dissociation constant *K*_*d*_ = *k*_off_*/k*_on_. Results were means ± SE (*n* = 4 for rA30 and rA60, and *n* = 6 for DNA).

### Electrophoretic mobility shift assay (EMSA)

A_59_ RNA (10 nM) and 0–500 nM Nab2-6xHis were incubated in polyadenylation buffer plus 5% (v/v) glycerol and 0.025% Orange G for 1.5 h at 30°C and then resolved on 6% native PAGE in His-MES buffer (38 mM L-histidine, 5.86 g/L MES hydrate at pH 6.4, determined at 25°C) at 300 V for 1 h at 4°C. The Atto680-labeled RNA was visualized with an Odyssey imager. Unbound (A_59_), bound monomeric (A_59_:Nab2), and bound dimeric (A_59_:2 × Nab2) complexes were quantified using ImageJ. Fractional saturation (*Y*) was calculated as

$$Y=\frac{\left(A_{59}:\mathrm{Nab}2)\;+\;2\times (A_{59}:2\times \mathrm{Nab}2\right)}{2\times (A_{59})\mathrm{tot}},$$

where [A_59_]_tot_ = 10 nM. This assumes that Nab2 has two binding sites on A_59_ RNA. The data points were fit in Origin to the Hill equation

$$\log\left(\frac{Y}{1-Y}\right)=n_{H}[\log(\mathrm{Nab}2)]-\log\left(K_{d}\right),$$

where *n*_*H*_ is the Hill coefficient and *K*_*d*_ is the dissociation constant. Note that the *K*_*d*_ derived from the *Y* intercept in a Hill plot is meaningless for interactions that do not occur with perfect cooperativity.

### Size exclusion chromatography multiangle light scattering (SEC-MALS)

Samples in polyadenylation buffer were incubated for 1 h at 30°C, spun at 10,000*g* for 10 min, and loaded (45–75 µL, 50–80 µg of protein) onto a Superdex 200 Increase 10/300 GL column (GE Healthcare). Sample compositions were as follows: 17 µM A59, 35 µM A30, 31 µM Nab2-6xHis, 15 µM Nab2-6xHis + 7.5 µM A59, 16.5 µM Nab2-6xHis + 4.125 µM A59, and 15 µM Nab2-6xHis + 15 µM A30. MALS was performed on a miniDAWN (Wyatt Technology) instrument with an Optilab refractive index (RI) detector and quasielastic light scattering (QELS) modules. The column and the HPLC detectors were placed in the cooling cabin with temperature ranging between 5°C and 7°C. MALS (including QELS) and RI detectors were placed outside the cabin and adjusted to 20°C. Data were processed with Astra V 7.3.2 software (Wyatt Technology). The *dn*/*dc* and UV extinction coefficient values for the protein and RNA used as an input in Astra are reported in Supplemental Table S1. Molecular weights were calculated by using the RI as the concentration source. For conjugate analyses, the RI and UV were used as concentration sources.

### Mass photometry

Samples were prepared in polyadenylation buffer, incubated for 10 min at room temperature, and recorded for 3 min on a Refeyn TwoMP Auto in manual mode with buffer-free focusing. Mass calibration used 66 kDa BSA and 150 or 300 kDa IgG. Event histograms were fit with Gaussians using Refeyn Discover MP software.

### Formaldehyde cross-linking

Nab2-6xHis (250–500 nM) was mixed with 0 or 100 nM RNA in polyadenylation buffer using the Sarstedt protein low-binding tubes and incubated for 1 h at 30°C. Formaldehyde solution (3%) was then added at a final concentration of 0.3% (v/v) for 10 min and quenched by adding 2 M glycine solution at a final concentration of 0.2 M for 5 min. Samples were transferred to a new tube and mixed with LDS and DTT, incubated for 10 min at 30°C, and run on NuPAGE 4%–12% Bis-Tris gels (Invitrogen). Atto680-labeled RNA was visualized with an Odyssey imager, followed by silver staining using Thermo Scientific Pierce silver stain kit.

### UV-cross-linking

Samples were prepared in polyadenylation buffer by incubating RNA (final concentration 25 nM) with CF IA/IB for 7 min at 30°C, and then CPF, Nab2-FKBP12/FRB, and either DMSO or rapamycin were added, followed by an additional 7 min incubation. The reactions were irradiated for 30 sec with 254 nm UV light and placed on ice. Samples were mixed with LDS sample buffer and DTT and resolved on NuPAGE 4%–12% Bis-Tris gels. RNA–protein cross-links were visualized via the Atto680 label using an Odyssey imager and then quantified with ImageJ.

### Yeast poly(A) tail length assays

Poly(A) tail length assays using RNase H/Northern detection of *HSP104* RNA 3′ ends after heat shock and mRNA export block in the *MEX67-AA* strain (*tor1-1 fpr1::NAT RPL13-2xFKBP12*::*TRP1 MEX67-FRB*::*kanMX6 MAT*α; Euroscarf) were conducted as reported previously by Turtola et al. (2021) and as detailed in the Supplemental Material. Cells were transformed with pESC-*URA3* (pPgal) plasmids carrying *NAB2* variants (see Supplemental Table S2).

### Viability assays and Nab2 protein levels

The viability of *NAB2* mutations was evaluated by transforming the *nab2*Δ*::HIS3* p(*NAB2*/*CEN, URA3*) *ura3 leu2 trp1 MAT*α strain (Marfatia et al. 2003) with p(*NAB2*/*CEN, LEU2*) plasmids (Supplemental Table S2). Transformed cells were grown overnight in SC-leu media. Cultures were adjusted to OD_600_ = 1, and 10-fold serial dilutions were spotted on the SC-leu or SC + 5-FOA (1 mg/mL) plates, followed by incubation at the indicated temperatures and imaging. To cure the p(*NAB2*/*CEN, URA3*) plasmid, cells were passaged twice on SC + 5-FOA, yielding *nab2*Δ*::HIS3* p(*NAB2*/*CEN, LEU2*) *ura3 leu2 trp1 MAT*α strains with the indicated *NAB2* mutations. These were cultured at 30°C in SC medium to an OD_600_ ∼ 0.6, and Nab2 protein levels were determined by Western blotting as described in the Supplemental Material.

## Supplemental Material

Supplement 1
