# Supplementary material for: A kinetic ruler controls mRNA poly(A) tail length
Source: Genes Dev. 2025 Nov 1;39(21-22):1377–94. doi: 10.1101/gad.352912.125 (PMC12581835; doi:10.1101/gad.352912.125)
Supplement: Supplement 1 [file Supplemental_Data_Gabs_et_al.pdf]

Supplemental information for

A kinetic ruler controls mRNA poly(A) tail length

Emilie Gabs, Emil Aalto-Setälä, Aada Välisaari, Anssi M. Malinen, Torben Heick Jensen, Stephen H. McLaughlin, Lori A. Passmore, Matti Turtola

SUPPLEMENTAL MATERIALS AND METHODS

DNA constructs

The cloning involving the pESC-URA (pPgal), p(*CEN*, *LEU2*) and pET28b was performed in DH5α or XL1 *E. coli* cells. A list of plasmids is in **Supplemental Table S2**. *NAB2* deletions and point mutations were first constructed in pESC-URA vectors. The N-terminal (ΔNTD) and C-terminal (ΔZnF5-7-CT and ΔCT) truncations were created by amplifying the included fragment of the coding region by PCR with primers containing overhangs with EcoRI sequence at the 5′end and the NotI site at the 3′end of the amplified sequence (see **Supplemental Table S3** for primer sequences and DNA templates used in the PCR reactions). The internal deletions were constructed in two rounds of PCR by first amplifying the N-terminal and C-terminal fragments separately with primers containing overhangs that created complementary sequences across the deleted region. These purified fragments were then used as a template in a second PCR reaction with primers that annealed to the N-terminal and C-terminal ends and contained overhangs with EcoRI and NotI sites, respectively. The ΔQQQP fragment was amplified using pAC1039 (Marfatia et al. 2003) as a template in PCR. The chimeric gene where ZnF1-7-CT region of *S. cerevisiae* Nab2 was replaced with ZnF1-5 of human ZC3H14 was constructed by fusing two fragments in a PCR reaction: a PCR-amplified N-terminal fragment of Nab2 (amino acid residues 1-255), and a DNA string fragment (Thermo Scientific/Gene Art) of human ZC3H14 (amino acid residues 594-736; codon optimized for expression in *S. cerevisiae*). The PCR products were cloned into the pESC-URA vector under the pGal10 promoter using EcoRI and NotI. In the indicated constructs (**Supplemental Table S2**) the fragment was cloned in-frame with the C-terminal Flag-tag sequence by omitting the stop codon from the 3′end overhang of the amplified sequence. *NAB2* point mutations were created by inverse PCR, amplifying the whole plasmid DNA, followed by DpnI digestion of the template plasmid, T4PNK phosphorylation of linear DNA ends, and blunt end ligation. The *NAB2* coding sequences were confirmed by Sanger DNA sequencing and the overall composition of the plasmids were analysed by restriction digestion.

The p(*CEN*, *LEU2*) parent plasmid was constructed by PCR-amplifying sequences 400 bp upstream and 400 bp downstream of the *NAB2* ORF with complementary overhangs using pAC1039 (Marfatia et al. 2003) as a DNA template, and inserted into HindIII and SacI-linearized pRSII415 vector (Chee and Haase 2012) through NEBuilder HiFi DNA Assembly (New England Biolabs). Subsequently, p(*NAB2*/*CEN*, *LEU2*) plasmids were generated by cloning the *NAB2* genes (digested from the pESC-URA plasmids) using the EcoRI and NotI sites between the upstream and downstream sequences. The A-tract variants were created by inverse PCR, as indicated above, using the p(*CEN*, *LEU2*) parent plasmid as a template. The NdeI-SacI fragments were then cloned into the p(*NAB2*/*CEN*, *LEU2*) plasmid to replace the wild-type autoregulatory sequence with variable A-tract sequences.

To construct the *E. coli* expression vectors, *NAB2* genes, originally cloned into the pESC-URA plasmids (see above), were PCR-amplified using primers with specific overhangs. The resulting PCR products were inserted into NcoI and NotI -linearized pET28b vectors through NEBuilder HiFi DNA Assembly. The pEG012 (ΔZnF5-7) was constructed by inverse PCR using pEG005 (Nab2 wild-type; pET28b) as a template and

primers that created the deletion (amino acids 390-485 deleted), followed by DpnI digestion of the template plasmid, T4PNK phosphorylation of linear DNA ends, and blunt end ligation. To construct the Nab2-FKBP12 and Nab2-FRB fusion proteins, the PCR-amplified fragments of human FKBP12 (amino acid residues 1-107) or FRB (amino acid residues 2021-2113 of mTOR, T2098L) containing appropriate overhangs were included in the assembly reactions.

Expression of CPF modules, CF IA and SII-Nab2 in insect cells

The *PFS2* and *REF2* genes encoded a C-terminal Twin-Strep (SII)-tag. *PCF11* gene encoded an N-terminal SII-tag and *RNA14* encoded an N-terminal 8xHis-tag. *NAB2* gene encoded an N-terminal SII-tag. Vectors (see **Supplemental Table S2**) carrying the polymerase module genes (*CFT1*, *YTH1*, *FIP1*, *PAP1*, *PFS2*-SII) (Hill et al. 2019), nuclease module genes (*YSH1*, *CFT2*, *MPE1*) (Kumar et al. 2021), phosphatase module genes (*PTA1*, *PTI1*, *SSU72*, *SWD2*, *GLC7*, *REF2*-SII) (Kumar et al. 2021), CF IA genes (8xHis-Rna14, Rna15, SII-Pcf11, Clp1) (Kumar et al. 2021), or SII-*NAB2* (Turtola et al. 2021) gene were transformed into chemically competent DH10EmBacY cells and bacmids were isolated as previously described (Bieniossek et al. 2008). *Spodoptera frugiperda Sf9* cell line was used for the expression of baculovirus-based recombinant proteins and complexes. For CPF modules and CF IA, the bacmid transfection (production of P1 virus) was performed in *Sf9* cells that were cultured to log phase in Sf-900 II SFM medium (Gibco) before being transferred to ExpiSF CD Medium (Gibco) at a density of 0.5 × 10^6 cells/ml. Fugene HD transfection reagent was used following the manufacturer’s protocol (Promega). The cultures were incubated at 27 °C and monitored daily for signs of infection. The supernatant containing the P1 virus was collected, sterile-filtered, and stored at 4 °C with the addition of 2% FBS for stabilization. To amplify the P2 virus, a 1:75 (v/v) dilution of the P1 virus was used to infect suspension culture of *Sf9* cells, which had been grown in Sf-900 II SFM and then transferred to ExpiSF medium at a density of 1.5 × 10^6 cells/ml. The infected cells were cultured at 27 °C 140 rpm for 72-96 hours before harvesting the P2 virus stock. Large-scale infections for protein expression were carried out in 500 ml cultures of *Sf9* cells at 2 × 10^6 cells/ml in Sf-900 II SFM or ExpiSF medium (27 °C, 140 rpm, 72-96 h) with 1:50 to 1:100 (v/v) P2 virus. The P2 viruses for nuclease module (1:60) and phosphatase module (1:75) were used for co-infection to express both modules simultaneously. Cells were harvested by centrifugation at 2,500 × g for 15 min and washed in PBS. Pellets were flash frozen in liquid nitrogen and stored at -80 °C. SII-Nab2 was expressed as described in (Turtola et al. 2021).

Expression of CF IB (6xHis-Hrp1) and Nab2-6xHis in E. coli

CF IB (6xHis-Hrp1) (Casañal et al. 2017) and Nab2-6xHis proteins were expressed in Xjb RIL *E. coli* cells transformed with plasmids detailed in **Supplemental Table S2**. The cells were grown in LB medium at 37°C. For Nab2-6xHis expression, the medium was supplemented with 0.1 mM $ZnCl_2$. IPTG (1 mM) and arabinose (0.1%, w/v) were added at an optical density (OD_{600}) of approximately 0.6, and the cells were harvested 3-5 hours later.

Purification and reconstitution of CPF

CPF polymerase module was purified as in (Rodríguez-Molina et al. 2022). Frozen pellet from 1 l culture was thawed in lysis buffer (50 mM Hepes-NaOH, pH 8.0, 300 mM NaCl, 1 mM TCEP) supplemented with cOmplete EDTA-free protease inhibitor cocktails (Sigma-Aldrich), and 1 ml BioLock biotin blocking solution (IBA Lifesciences). Cells were lysed by sonication and lysates were cleared by centrifugation at 40 000 × g for 15 min at 4 °C. The clarified lysate was bound in batch to 3 ml Strep-Tactin Sepharose beads (IBA Lifesciences) for 1 h on a rotating platform at 4 °C and subsequently transferred to gravity-flow column. Beads were washed extensively with lysis buffer, and subsequently incubated for 20 minutes with the elution buffer (50 mM Hepes-NaOH, pH 8.0, 150 mM NaCl, 1 mM TCEP and 1.2 mg/ml desthiobiotin) before eluting. The eluted sample was filtered and loaded onto a 1 ml Hitrap Q anion exchange chromatography column that was

equilibrated using buffer that contained 20 mM Hepes-NaOH, pH 8.0, 150 mM NaCl, 0.5 mM TCEP. The polymerase module was eluted using a NaCl gradient from 150 to 600 mM over 40 CV. The fractions containing all polymerase module subunits were pooled, concentrated using a 100 kDa molecular weight cut-off (MWCO) concentrators and snap-frozen in liquid N_2. The purification of the CPF nuclease-phosphatase module was carried out as described above, with different buffers. Here, the lysis buffer contained 50 mM Hepes-KOH, pH 8.0, 150 mM KCl, 0.5 mM magnesium acetate and 0.5 mM TCEP. The anion exchange chromatography was carried out using a 5 ml ResourceQ column (Cytiva) and a lysis buffer-based NaCl gradient from 150 to 500 mM over 15 CV. The complete CPF complex was reconstituted from separately purified polymerase module and the nuclease-phosphatase modules as in (Rodríguez-Molina et al. 2022). The CPF was reconstituted at final 10 µM by mixing in 1:1 molar ratio preparations of polymerase module and nuclease-phosphatase modules and separating the mixture in a Superose 6 Increase column. Peak fractions were analysed by SDS-PAGE and the fractions containing stoichiometric ratios of individual subunits were pooled, concentrated and snap-frozen in liquid N_2 for storage.

Purification of CF IA

CF IA was purified according to (Kumar et al. 2021). Frozen pellet from 2 l culture (~40 ml) was thawed into a total volume of 150 ml in lysis buffer (50 mM Hepes-NaOH, pH 7.9, 250 mM NaCl, 5% (w/v) glycerol, 0.5 mM TCEP) supplemented with 2 µg/ml DNase I (Thermo Scientific), 4 × cOmplete EDTA-free protease inhibitor cocktail tablets (Sigma-Aldrich), and 1 ml BioLock biotin blocking solution (IBA Lifesciences). Cells were lysed by sonication and lysates were cleared by ultracentrifugation at 42 000 × rpm in a Beckmann 50.2 Ti rotor for 45 min at 4 °C. The clarified lysate was bound in batch to 3 ml Strep-Tactin Sepharose beads (IBA Lifesciences) for 1 h on a rotating platform at 4 °C and subsequently transferred to a gravity-flow column. Beads were washed with 150 ml of lysis buffer, and subsequently incubated for 20 minutes with the lysis buffer supplemented with 1.2 mg/ml desthiobiotin before eluting. The protein sample was loaded onto a 5 ml HiTrap Heparin column (Cytiva), eluted using a NaCl gradient from 250 mM to 1000 mM over 15 CV, and the peak fractions were concentrated using a 10 kDa MWCO concentrator. The concentrated protein was further purified through Superdex 200 Increase size exclusion chromatography column using a buffer that contained 20 mM Hepes-NaOH, pH 8.0, 250 mM NaCl, 0.5 mM TCEP. The fractions containing all CF IA subunits were pooled and concentrated before being snap-frozen in liquid N_2 for storage.

Purification of CF IB (6xHis-Hrp1)

CF IB was purified as in (Casañal et al. 2017). Cell pellets were resuspended in Hrp1 lysis buffer (50 mM Hepes-KOH, pH 8.0, 300 mM NaCl, 0.5 mM TCEP, 20 mM imidazole) supplemented with 10% (w/v) glycerol, 2 µg/ml DNase I (Thermo Scientific), 2 µg/ml RNase A (Thermo Scientific) and cOmplete EDTA-free protease inhibitor cocktail tablets (Sigma-Aldrich). The cells were lysed by sonication on an ice bath, using 30-second on/off cycles for a total of 15 min. The lysate was then centrifuged at 20000 rpm for 30 min at 4 °C using a Beckmann JA-25.50 rotor. The cleared lysate was incubated with Ni-NTA beads on a rotating platform at 4 °C for 1.5 hours and subsequently transferred to gravity-flow column. The column was washed sequentially with the Hrp1 lysis buffer and wash buffer (50 mM Hepes-KOH, pH 8.0, 300 mM NaCl, 0.5 mM TCEP, 30 mM imidazole) before the Hrp1 protein was eluted with elution buffer (50 mM Hepes-KOH, pH 8.0, 300 mM NaCl, 0.5 mM TCEP, 300 mM imidazole). The eluate was directly loaded onto a 5 ml HiTrap Heparin column (Cytiva), equilibrated with a buffer containing 20 mM Hepes-KOH, pH 8.0, 100 mM NaCl, 0.5 mM TCEP. The protein was eluted using a NaCl gradient from 100 to 1000 mM over 10 CV. Fractions containing Hrp1 were pooled, concentrated with a 10 kDa MWCO concentrator (Pierce), and further purified by size exclusion chromatography in Superdex 200 Increase column (Cytiva) using a buffer that contained 20 mM Hepes-KOH, pH 8.0, 250 mM NaCl, 0.5 mM TCEP, and snap-frozen in liquid N_2.

Purification of Nab2 (wild-type and mutants)

SII-tagged Nab2 expressed in insect cells was purified as in (Turtola et al. 2021), with modifications. Cell pellet from 2 l culture was resuspended in lysis buffer (50 mM Tris-HCl, pH 8.0, 50 mM NaCl, 2 mM $MgCl_2$, 0.1 mM $ZnCl_2$, 1 mM TCEP) supplemented with 4 µg/ml DNaseI (Sigma-Aldrich), 4 µg/ml RNaseA and 42 U/ml Benzonase (Sigma-Aldrich). The cells were lysed using sonication, followed by centrifugation at 18000 rpm for 15 min at 4 °C using a Beckmann JA-25.50 rotor. The cleared lysate was supplemented with BioLock biotin blocking solution (IBA Lifesciences) and incubated for 20 additional min at 20 °C for completing nuclease digestion. NaCl concentration of the lysate was adjusted to 200 mM before incubating the lysate with the Strep-Tactin beads for 2.5 h under rotation at 4 °C. The beads were then moved to a gravity flow column and washed with wash buffer (20 mM Tris-HCl, pH 8.0, 0.1 mM $ZnCl_2$, 0.5 mM TCEP) containing 200 mM NaCl and 0.2% Tween-20, then with wash buffer containing 300 mM NaCl, wash buffer with 500 mM NaCl, wash buffer with 75 mM NaCl, and finally eluted with wash buffer containing 75 mM NaCl and 1.2 mg/ml desthiobiotin. The eluate was combined with three volumes of buffer A (20 mM MES, pH 6.5, 75 mM NaCl, 0.1 mM $ZnCl_2$, 0.5 mM TCEP), loaded to a ResourceS column (Cytiva) and eluted using a NaCl gradient. Peak fractions were combined and concentrated using 10 kDa MWCO concentrator before snap-freezing aliquots in liquid N_2.

To purify the Nab2-6xHis proteins expressed in *E. coli*, the cell pellets were resuspended in Nab2 lysis buffer (50 mM Hepes-KOH, pH 7.4, 500 mM NaCl, 5% (w/v) glycerol, 1 mM TCEP, 0.2% Tween-20 (v/v), 0.1 mM EDTA, 0.1 mM $ZnCl_2$) supplemented with cOmplete EDTA-free protease inhibitor cocktail tablets (Sigma-Aldrich). The cells were lysed using sonication on an ice bath, applying 30-second on/off cycles for a total of 15 min. The lysate was then centrifuged at 20000 rpm for 30 min at 4 °C using a Beckmann JA-25.50 rotor. The cleared lysate was supplemented with 20 mM imidazole and loaded on a GoBio Zn-IDA column (Bio-Works) to capture the 6xHis-tagged Nab2. The column was washed with 10 column volumes (CV) of the Nab2 lysis buffer containing 20 mM imidazole, followed by 6 CV of lysis buffer containing 50 mM imidazole. The protein was then eluted using lysis buffer supplemented with 200 mM imidazole. The first 15 ml of the elution were mixed with 35 ml of Buffer A (20 mM Hepes-KOH, pH 7.4, 5% glycerol, 0.5 mM TCEP, 0.1 mM EDTA, 0.1 mM $ZnCl_2$), and the mixture was filtered before loading onto to a 5 mL GoBio MiniS column (Bio-Works) for cation exchange chromatography. The column was washed with 5 CV of Buffer A, followed by a steep NaCl gradient from 150 to 1000 mM over 4.2 CV. 6xHis-Nab2 began eluting at ~400 mM NaCl. Eight earliest fractions containing Nab2 (total 8 ml) were pooled and concentrated to 1 ml using 10 kDa MWCO concentrators (Pierce). The concentrated sample was centrifuged at 10000 × g for 10 min at 4 °C before 500 µl was injected into a Superdex 200 column (Cytiva) equilibrated with gel filtration buffer (20 mM Hepes-KOH, pH 7.4, 500 mM NaCl, 0.5 mM TCEP, 0.1 mM EDTA, 0.1 mM $ZnCl_2$). Peak fractions of the gel filtration run containing Nab2, as detected by SDS-PAGE analysis, and displaying lowest A_{260}/A_{280}-ratios were pooled and concentrated further in 10 kDa MWCO concentrator before snap-freezing aliquots in liquid N_2.

The purification of Nab2 ΔRGG was performed otherwise as described above, with the exception that the buffers used for MiniS cation exchange and the elution step from the Zn-IDA column were prepared with MES buffer (pH 6.0) instead of Hepes buffer (pH 7.4).

The purification of Nab2 ΔZnF1-4 was carried out as described above up to the MiniS cation exchange step. Due to a high nucleic acid content in the MiniS eluate, the peak fractions were pooled and diluted 1:10 in Buffer A containing 120 mM NaCl and 2 mM $MgCl_2$. The diluted sample was treated overnight with 50 U/ml benzonase (Pierce) at 4°C. Afterwards, benzonase and degraded nucleic acids were removed by a second MiniS cation exchange chromatography step, performed as described above. The peak fractions were then pooled, concentrated, and snap-frozen for storage.

The purification of Nab2 ΔZnF5-7, which has a lower isoelectric point than the wild-type protein, followed the same procedure as the wild-type, except that a MiniQ column (Bio-Works) was used instead of a MiniS column during the second purification step to perform anion exchange chromatography using the same buffers.

RNA oligonucleotides

Oligonucleotide sequences and information are provided in **Supplemental Table S4**. RNA oligonucleotides were purchased from Eurogentec. Certain RNAs were prepared in-house using splinted ligation. This method employed a complementary DNA splint to align the 3′-OH and 5′-phosphate termini of two RNA strands, facilitating their ligation by T4 DNA ligase (Thermo Scientific). The reaction products were resolved on denaturing urea-polyacrylamide gels, detected through the fluorescence of 5′-Atto680 labels, excised from gel and purified using ZR small-RNA PAGE Recovery Kit (Zymo Research).

Determination of nuclear HSP104 mRNA poly(A) tail lengths from strains expressing mutant Nab2 proteins

Poly(A) tail length assays were conducted as reported in (Turtola et al. 2021). *MEX67-AA* strain (*tor1-1 fpr1::NAT RPL13-2xFKBP12*::*TRP1 MEX67-FRB*::*kanMX6 MATalpha*; Euroscarf) was transformed by the pESC-URA (pPgal) plasmids containing different *NAB2* constructs (**Supplemental Table S2**). Cells were cultured in synthetic complete (SC) medium lacking uracil and containing 2% raffinose and 0.1% glucose (SC-ura/raf). Three hours before heat shock 2% galactose was added to cell cultures which had an OD_{600} ~0.5 to induce the protein expression from pESC-URA plasmids. Protein extracts, used for quantifying Nab2 expression levels by western blotting, were prepared from cells harvested 3 hours after adding the galactose. Cultures kept at 25˚C were heat-shocked by rapidly mixing an equal volume of identical media pre-heated to 51˚C and transferring the cultures to a 38 ˚C water bath. Rapamycin (final concentration 1 µg/ml; Life Technologies) was added to cultures 5 min before heat shock. Cells for RNA analysis were harvested by mixing equal volumes of culture and 96% ethanol pre-cooled on dry ice and centrifuging at 3000 × g for 3 min at 4 ˚C.

RNA extraction was performed with the hot phenol method. Briefly, a frozen cell pellet was resuspended in TES-buffer (1% SDS, 5 mM EDTA, 10 mM Tris-HCl, pH 7.5) and an equal volume of 0.1 M citrate buffered phenol pH 4.3 (Sigma P4682). The mix was incubated at 65 ˚C for 40 min under 1400 rpm shaking, followed by centrifugation at 16000 × g for 10 min at 4 ˚C. The aqueous phase was extracted again with acidic phenol at 65 ˚C for 20 min, and twice with chloroform at room temperature. RNA was precipitated with ethanol (final 70%) and 60 mM LiCl over-night. The pellet was washed with EtOH and resuspended in water.

RNaseH northern blotting was performed as reported in (Turtola et al. 2021). Briefly, 20 µg of total RNA was combined with 2 µM DNA oligonucleotide (DL163 (Libri et al. 2002); oligonucleotide sequences are listed in **Supplemental Table S4**) complementary to the *HSP104* 3′ region in annealing buffer (50 mM Tris-HCl, pH 8.3, 50 mM KCl) in a total volume of 12 µl. The mix was incubated at 85 ˚C for 2 min and slowly cooled to 37 ˚C. Subsequently, annealing reactions were supplemented with 8 µl mix pre-heated to 37 ˚C and containing 2.5 U of RNaseH (New England Biolabs), 2.5 × RNaseH reaction buffer (1× buffer: 50 mM Tris-HCl, 75 mM KCl, 3 mM $MgCl_2$, 10 mM DTT, pH 8.3), 25 mM DTT and 4 U RiboLock RNase inhibitor (Thermo Scientific). The mix was incubated at 37 ˚C for 30 min, followed by addition of 100 µl absolute ethanol and 20 µl of solution containing 600 mM sodium acetate (pH 5.3), 10 mM EDTA, 5 µg tRNA (Roche 28473522) and 5 µg glycogen. RNA was then precipitated at -20 ˚C. The pellet was washed with 70% ethanol and resuspended in RNA loading buffer (formamide, 10 mM Tris-HCl pH 8.0, 5 mM EDTA, 0.02% xylene cyanol). Samples were separated on 6% urea-polyacrylamide gels by electrophoresis, transferred to Hybond-N+ membrane (GE Healthcare RPN203B), hybridized over-night at 50 ˚C with 5′ terminally ^{32}P-labelled DNA oligonucleotide probes DL164 (Libri et al. 2002); and MS618 (Schmid et al. 2015) in ULTRA-Hyb Oligo Hybridization buffer

(Invitrogen AM8663), washed 4 times with 2 × SSC (300 mM NaCl, 30 mM trisodium citrate, pH 7.0) containing 0.5% SDS, each time rotating for 30 min at 42 ˚C, and exposed to phosphorimager screen. Images were processed and quantitated with ImageJ software. The indicated poly(A) tail lengths were approximated from a DNA size marker.

Western blotting

Cells equivalent to 5 OD units were lysed by vortexing with glass beads in 8 M urea for 5 minutes at 4 ˚C, followed by incubation at 95 ˚C for 10 minutes. Lysate was clarified by centrifugation. Total protein concentration was determined by a Bradford assay, and equal amounts protein were loaded onto an SDS-PAGE gel. Western blotting was carried out with standard procedures using antibodies for Nab2 (HL831 3F2; (Anderson et al. 1993)), Rpb3 (1Y26; Abcam), and FlagM2 (F1804; Sigma-Aldrich), which were detected using either HRP-conjugated secondary anti-mouse polyclonal goat immunoglobulins (Dako) or Alexa Fluor 680-conjugated secondary anti-mouse cross-adsorbed secondary antibody (A21057; Invitrogen).

SUPPLEMENTAL FIGURES

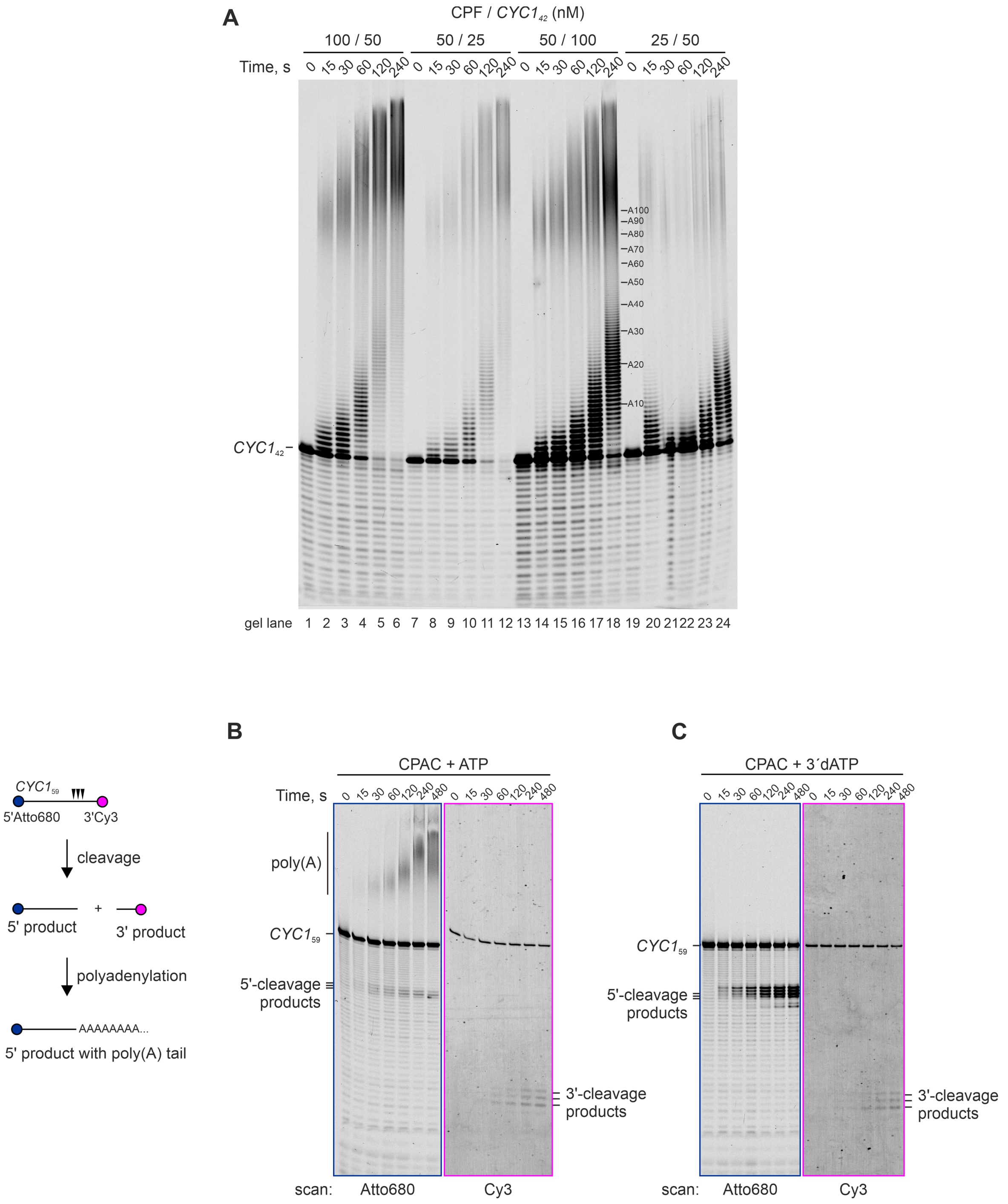

Supplemental Figure S1. (*A*) The effect of RNA and CPF concentrations on polyadenylation. The concentrations of CF IA (500 nM), CF IB (500 nM) and ATP (2 mM) were the same in all reactions. (*B*) Cleavage and polyadenylation. A dual-labelled *CYC1*$_{59}$ RNA substrate with 5′Atto680 and 3′Cy3 dyes was used for tracking the 5′ and 3′ products. The RNA was pre-incubated with CF IA and CF IB, before the start of the reactions by the simultaneous addition of CPF and ATP. The scans of the same gel area are shown for Atto680 (*left*; blue outline) and Cy3 (right; magenta outline) channels. (*C*) As in *B* but 3′dATP was included in the reactions to prevent polyadenylation.

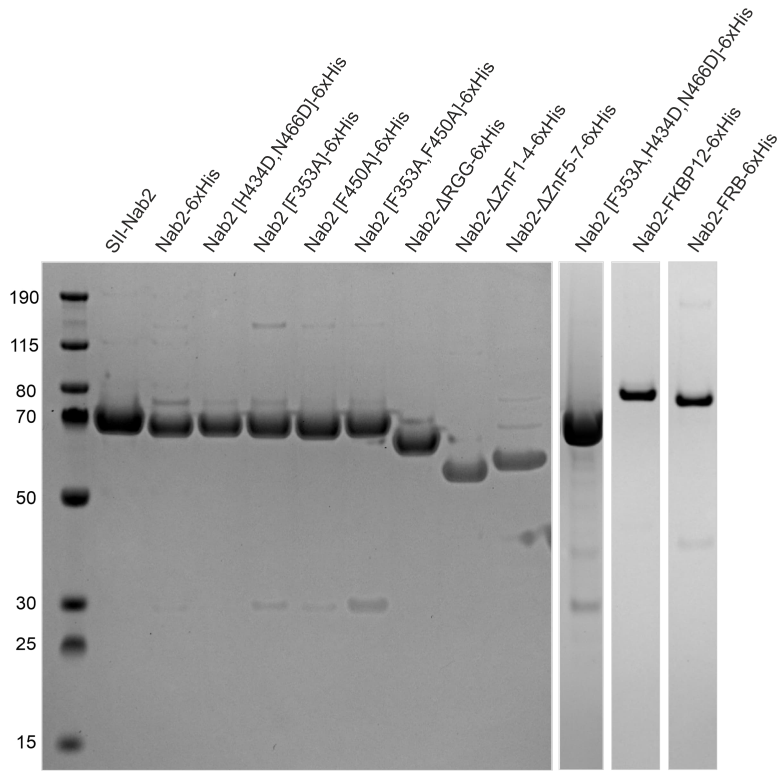

Supplemental Figure S2. Purified Nab2 proteins used in Figures 2-6 separated by SDS-PAGE and visualised by Coomassie staining. SII-Nab2 was expressed in *Sf9* insect cells. Other proteins were expressed in *E. coli*.

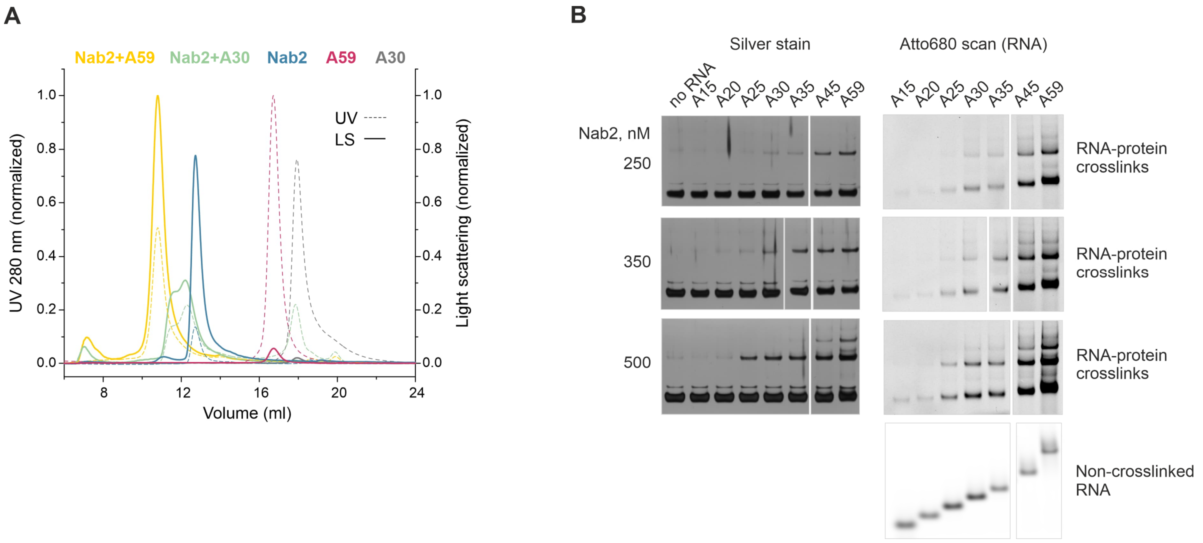

Supplemental Figure S3. (*A*) UV (280 nm) and light scattering traces of the SEC-MALS analyses for A_{59} (magenta), A_{30} (light grey), Nab2 (blue) and Nab2 mixed with either A_{59} (yellow) or A_{30} (green). Light scattering intensities are displayed (y-axis on the right side) along the elution volume (x-axis). The absorbance is displayed by dashed lines with corresponding colours (y-axis on the left side). All absorbance values were normalized to the highest value in the A_{59} RNA sample. All light scattering values were normalized to the highest value in the Nab2+A_{59} RNA sample. (*B*) Formaldehyde crosslinking of Nab2 (250, 350, 500 nM) with Atto680-labelled poly(A) RNAs (100 nM) of varying lengths. The panels were cropped together from two gels exposed at the same time.

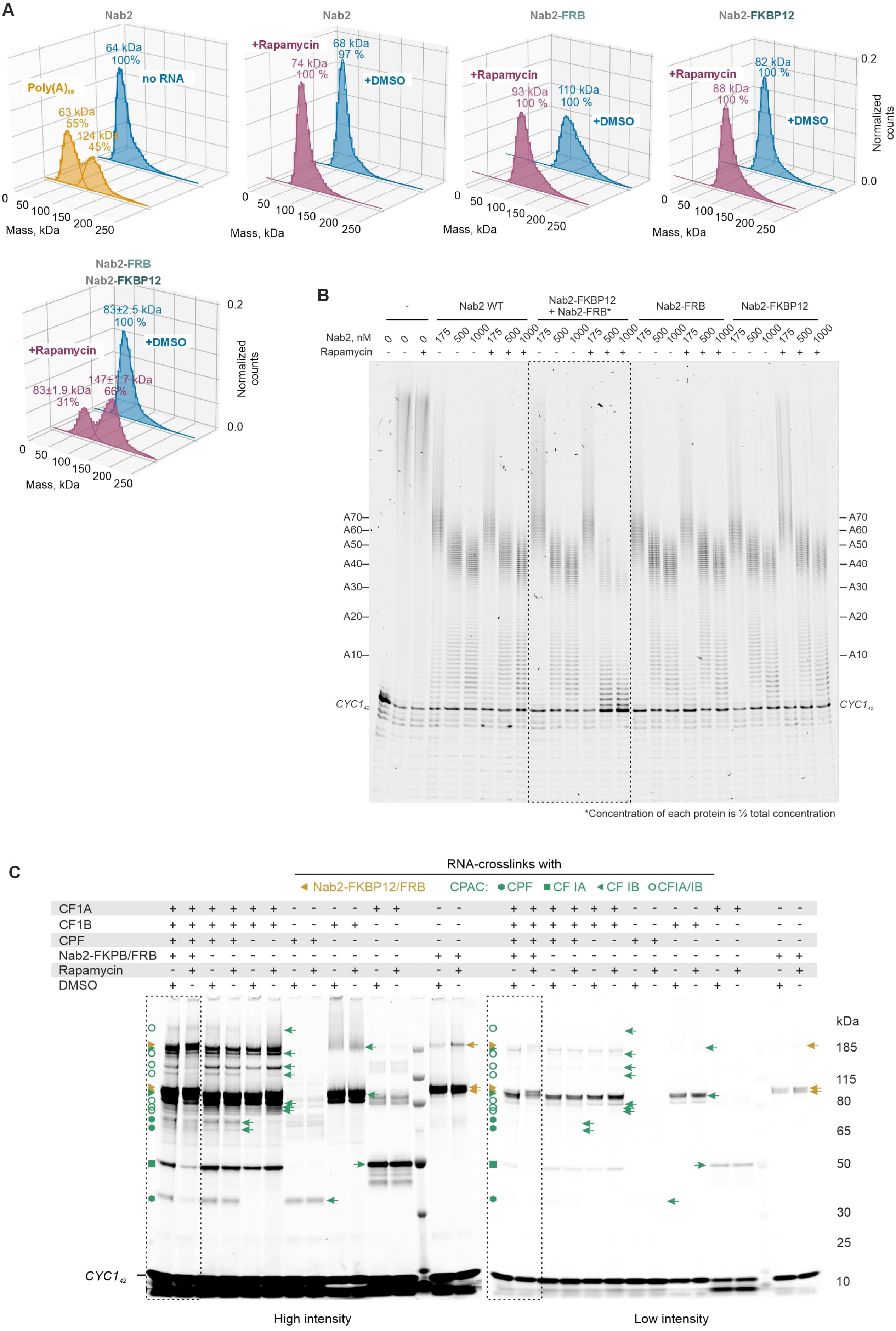

A
Nab2
Poly(A)59
no RNA
64 kDa
100%
63 kDa
55%
124 kDa
45%
+Rapamycin
+DMSO
68 kDa
97 %
74 kDa
100 %
Nab2-FRB
93 kDa
100 %
110 kDa
100 %
Nab2-FKBP12
82 kDa
100 %
88 kDa
100 %
Mass, kDa
Normalized counts
Nab2-FRB
Nab2-FKBP12
83±2.5 kDa
100 %
83±1.9 kDa
31%
147±1.7 kDa
66%
B
Nab2 WT
Nab2-FKBP12 + Nab2-FRB*
Nab2-FRB
Nab2-FKBP12
Nab2, nM
Rapamycin
A70
A60
A50
A40
A30
A20
A10
CYC1_42
*Concentration of each protein is ½ total concentration
C
RNA-crosslinks with
Nab2-FKBP12/FRB
CPAC: CPF CF IA CF IB CFIA/IB
CF1A
CF1B
CPF
Nab2-FKPB/FRB
Rapamycin
DMSO
kDa
185
115
80
65
50
30
25
10
CYC1_42
High intensity
Low intensity

Supplemental Figure S4. (*A*) Mass photometric analysis of the effects of DMSO and rapamycin (1 µM) for the oligomerization state of Nab2, Nab2-FKBP12 and Nab2-FRB. The total concentration of Nab2 in all experiments was 250 nM. The combination of Nab2-FKBP12 and Nab2-FRB (each 125 nM) is the same as shown in Fig. 4B. Nab2 (250 nM) with A_{59} (50 nM) on the left is shown as a positive control for RNA-dependent dimerization of Nab2. The gaussian fits of the count-normalized histograms are shown as lines with the peak positions (in kDa) and the percentage of total peak counts displayed above. (*B*) The effects of rapamycin and the Nab2-FRB and Nab2-FKBP12 fusion proteins (added individually or together) on $CYC1_{42}$ polyadenylation by the CPAC. Nab2 proteins were incubated with DMSO (-rapamycin) or with 1 µM rapamycin before being added to the polyadenylation reactions together with ATP. The reactions were stopped after 4 minutes. The marked gel area is the same as displayed in Fig. 4C. (*C*) SDS-PAGE of UV-induced RNA-protein crosslinks, detected via Atto680 fluorescence from the $CYC1_{42}$ RNA (25 nM). High- and low-intensity scans of the same gel are shown. When included, reactions were performed with CPF (75 nM), CF IA/IB (500 nM each), Nab2-FKBP12/FRB (500 nM each) and either DMSO or rapamycin (1 µM). Crosslinked RNA-protein adducts corresponding to Nab2-FKBP12/FRB and CPAC subunits are marked with gold and green symbols, respectively, and their assignment is highlighted with arrows on the gel images. The marked gel areas are the same as displayed in Fig. 4E.

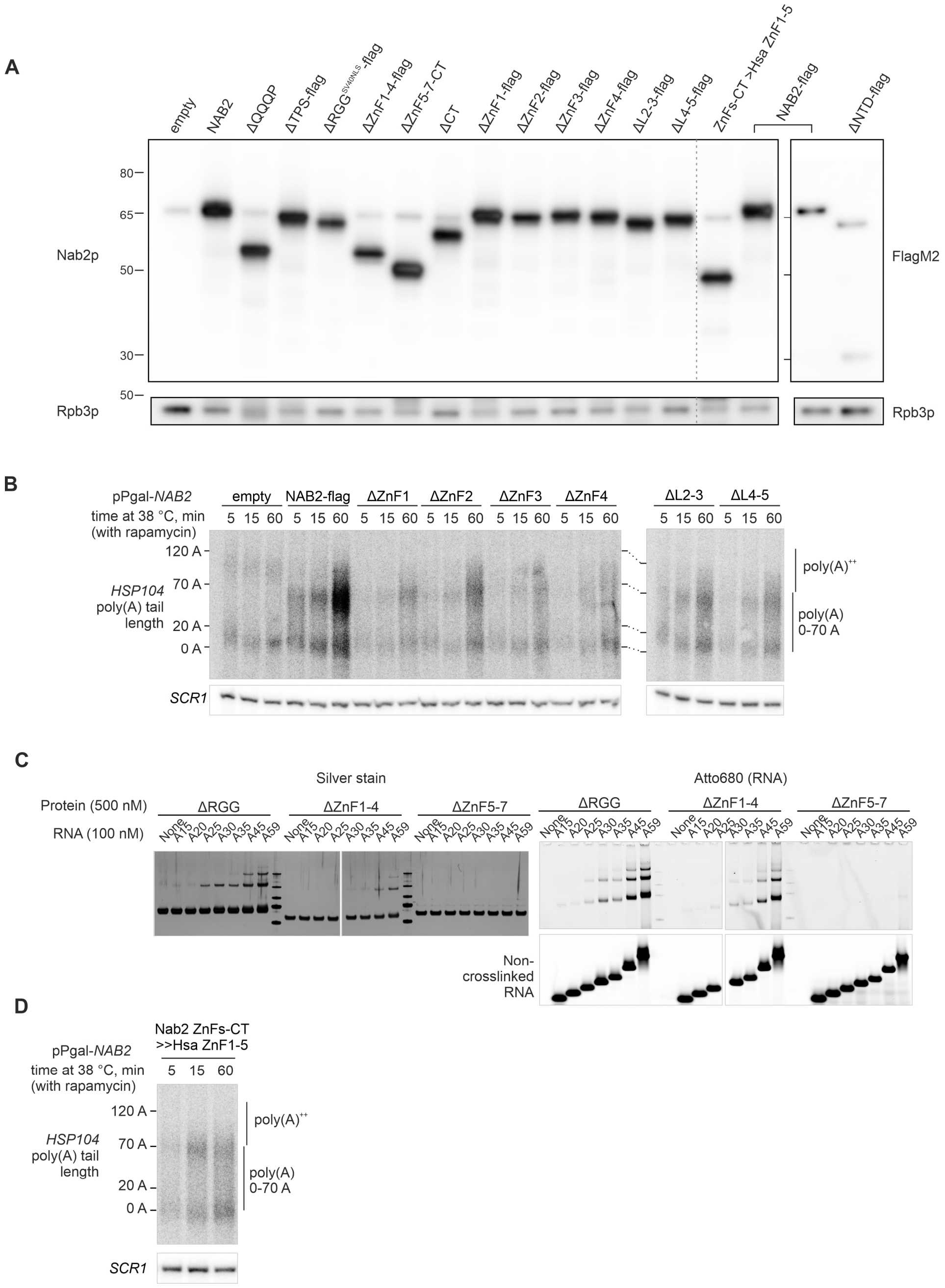

Supplemental Figure S5. (*A*) Western blot analysis of the Nab2 and Rpb3 protein levels in protein extracts from *MEX67-AA* cells over-expressing different Nab2 mutant variants used in Fig. 5B, Supplemental Fig. 5B and Supplemental Fig. 5D. The upper left panels were probed with an anti-Nab2 antibody (Anderson et al. 1993) that binds to the NTD. The ΔNTD-flag variant, and for comparison the wild-type Nab2-flag, were detected with an anti-FlagM2 antibody (upper right panel). Rpb3 was detected afterwards from the same membranes that were cut at the 50 kDa marker. Note that the endogenously expressed Nab2 protein is visible as a faint band migrating at the level of the 65 kDa marker. (*B*) Nuclear *HSP104* mRNA poly(A) tail lengths in cells expressing additional mutant variants of Nab2. See Fig. 5A-B for experimental details. (*C*) Additional data for Fig. 5D showing the silver stained and Atto680 scans of formaldehyde crosslinked Nab2 truncation mutants (500 nM) incubated with Atto680-labelled poly(A) RNAs (100 nM) of varying lengths. The panels were cropped together from two gels that were simultaneously exposed. (*D*) Nuclear *HSP104* mRNA poly(A) tail lengths in cells expressing a chimeric protein where the ZnF1-7-CT region of *S. cerevisiae* Nab2 is replaced by ZnF1-5 of human ZC3H14.

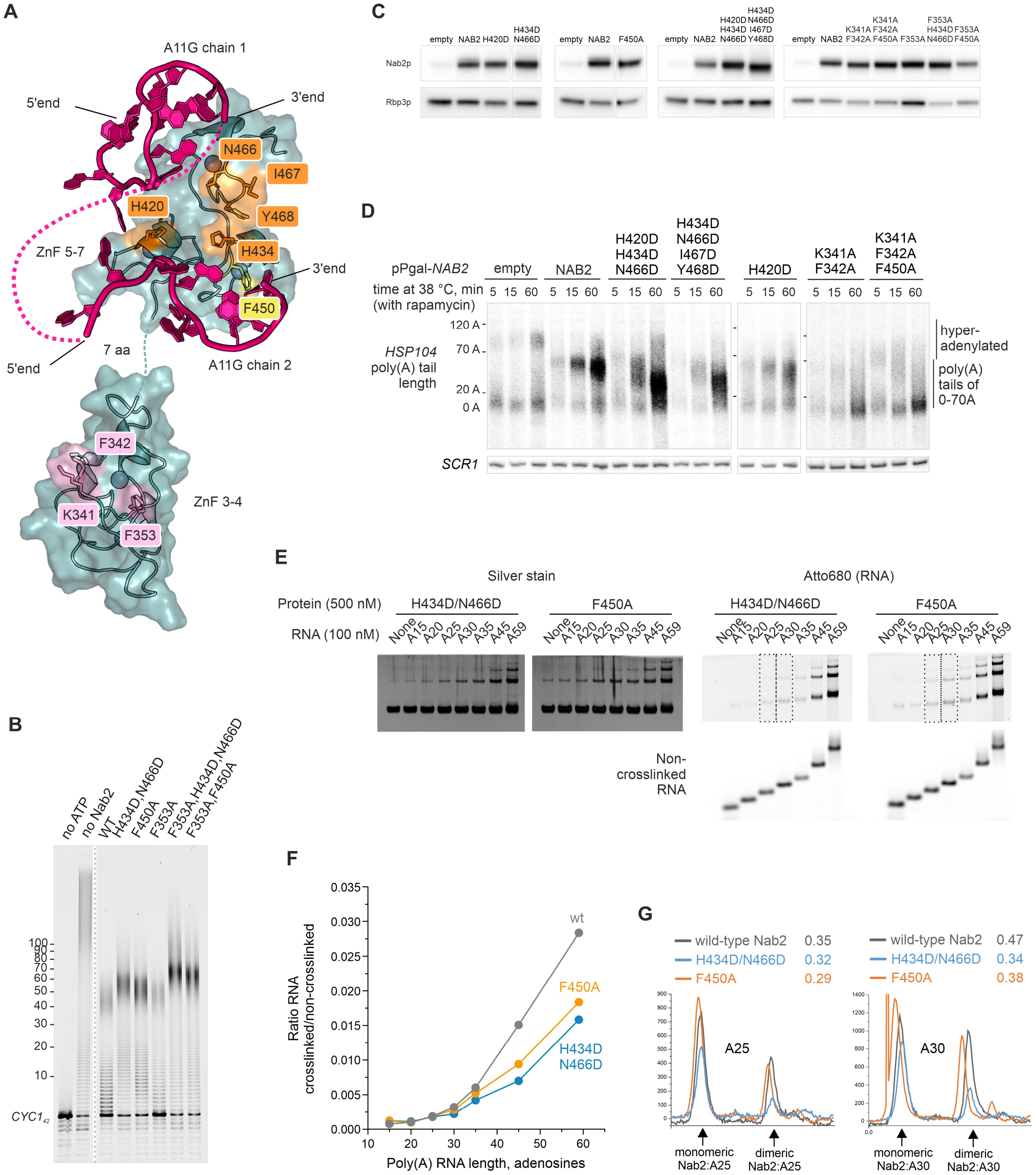

Supplemental Figure S6. (*A*) A composite model of Nab2 showing one ZnF5-7 domain bound to two chains of $A_{11}G$ RNA (*top*, PDB 5L2L; (Aibara et al. 2017)) and one ZnF3-4 domain (*bottom*, PDB 3ZJ2; (Martínez-Lumbreras et al. 2013)). The second ZnF5-7 domain is removed in order to display the dimeric interface surface. The protein residues mutated in this study are shown as sticks. The mutated dimer interface residues are highlighted in orange and the mutated residues interacting directly with RNA are highlighted in ZnF6 in yellow (F450) or in ZnF3 in pink (K341, F342, F353). The 5′ and 3′ ends of the RNAs are indicated. The dotted magenta line depicts a hypothetical path to connect two ends by an intervening RNA chain. (*B*) The effect of purified Nab2 point mutants (1000 nM) on CPAC-mediated *CYC1*$_{42}$

polyadenylation. (*C*) Western blot analysis of the Nab2 and Rpb3 protein levels in protein extracts from *MEX67-AA* cells over-expressing different Nab2 mutant variants used in Fig. 6C and Supplemental Fig. 6D. Gel panels with narrow spacing were cropped from the same gels. (*D*) Nuclear *HSP104* mRNA poly(A) tail lengths in cells expressing mutant variants of Nab2. See Fig. 5A-B for experimental details. (*E*) formaldehyde crosslinked Nab2 point mutants (500 nM) incubated with Atto680-labelled poly(A) RNAs (100 nM) of varying lengths. The signal intensity scans from the dashed areas are shown in *G*. (*F*) Quantification of poly(A) RNA-Nab2 crosslinks from *E*. The wild-type Nab2 (500 nM) data presented in Fig. 3E is replicated here. (*G*) Signal intensity scans of H434D/N466D and F450A mutants crosslinked with A25 and A30 RNAs. The corresponding scans of wild-type Nab2 crosslinks are shown for comparison. The intensities of the crosslinked RNAs were normalized to the total intensities of the non-crosslinked RNAs. The ratios of multimeric vs. total RNA-protein crosslinks are shown on top.

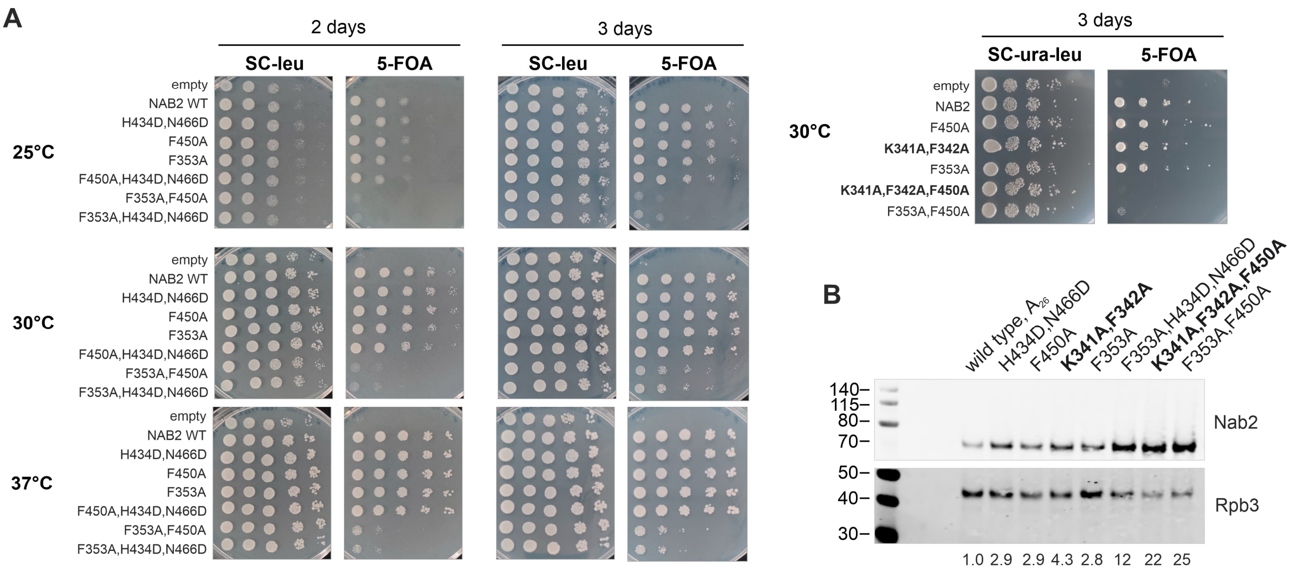

Supplemental Figure S7. (*A*) Growth assays for the *Δnab2::HIS3* pURA3/*NAB2* strain transformed by the p(*NAB2*/*CEN*, *LEU2*) plasmids carrying mutations in *NAB2*. The cells grown in SC medium lacking leucine (SC-leu), or uracil and leucine (SC-ura-leu) were adjusted to OD_{600}=1, 10-fold serial dilutions were spotted on the SC-leu (or SC-ura-leu) and SC + 5-FOA agar plates incubated at 25, 30 or 37 °C, and imaged at the indicated times. (*B*) Western blot analysis of the Nab2 and Rpb3 protein levels in the *Δnab2::HIS3* p(*NAB2*/*CEN*, *LEU2*) strains bearing the indicated mutations in *NAB2*. The mean of Nab2/Rpb3-ratios from two experiments normalized to the wild-type is shown below.

SUPPLEMENTAL TABLES

Supplemental Table S1. Size exclusion chromatography-multiangle light scattering data

**Protein**	Nab2-6xHis*	Nab2-6xHis*	Nab2-6xHis*	Nab2-6xHis*	-	-
**RNA**	A59**	A59**	A30***	-	A59**	A30***
Molar ratio (protein:RNA) in the sample	2:1	4:1	2:2	n/a	n/a	n/a
**MW based on refractive index (RI)**						
MW (kDa)	135.3	143.9	82.2	59.4	19.2	11.2
**Conjugate analysis, MW based on RI and UV extinction coefficient**						
MW (kDa)	135.6	144	83.1	n/a	n/a	n/a
MW Protein (kDa)	119.3	128	68.9	n/a	n/a	n/a
MW Modifier (kDa)	16.3	15.7	14.3	n/a	n/a	n/a

*Mr 59.143 kDa, dn/dc [ml/g] 0.185, UVext [ml/(mg×cm)] 0.224
**Mr 19.441 kDa, dn/dc [ml/g] 0.18, UVext [ml/(mg×cm)] 11.6
***Mr 9.894 kDa, dn/dc [ml/g] 0.18, UVext [ml/(mg×cm)] 5.90

Supplemental Table S2. Plasmids

**Identifier**	**Original name**	**Description**	**Source**
**Baculovirus integration vectors**			
CPF polymerase module	P20-3	pBIG1a (CFT1 N997D, PFS2-SII, YTH1, FIP1, PAP1)	Hill et al. 2019
CPF nuclease module	P20-6	pBIG1b (CFT2, YSH1, MPE1)	Kumar et al. 2021
CPF phosphatase module	P27-37, clone AC2	pBIG2ab (SSU72, PTI1, GLC7, REF2-SII, SWD2, PTA1)	Kumar et al. 2021
CF IA	P20-24	pBIG1c (8xHisRNA14, RNA15, SII-PCF11, CLP1)	Kumar et al. 2021
Nab2	P21-63	pACEBac1 (SII-NAB2)	Turtola et al. 2021
***E. coli* expression plasmids**			
Nab2	pEG005	pET28b C-terminal 6xHis	This study
Nab2-FKBP12	pEG025	pET28b C-terminal FKBP12 (H. sapiens FKBP12 residues 1-107), C-terminal 6xHis	This study
Nab2-FRB	pEG026	pET28b C-terminal FRB (H. sapiens mTOR residues 2021-2113, T2098L), C-terminal 6xHis	This study
Nab2_ΔRGG	pEG007	pET28b [201-255 deleted], C-terminal 6xHis	This study
Nab2_ΔZnF1-4	pEG006	pET28b [262-389 deleted], C-terminal 6xHis	This study
Nab2_ΔZnF5-7	pEG012	pET28b [390-485 deleted], C-terminal 6xHis	This study
Nab2_N466D,H434D	pEG008	pET28b N466D,H434D, C-terminal 6xHis	This study
Nab2_F450A	pEG009	pET28b F450A, C-terminal 6xHis	This study
Nab2_F353A	pEG010	pET28b F353A, C-terminal 6xHis	This study
Nab2_F353A,H434D,N466D	pEG017	pET28b F353A,H434D,N466D, C-terminal 6xHis	This study
Nab2_F353A,F450A	pEG011	pET28b F353A,F450A, C-terminal 6xHis	This study
CF IB	P2-43	pOPINB 6xHis-HRP1	Casañal et al. 2017
NAB2* in pPgal plasmid			
empty	pESC-URA	empty plasmid	Stratagene
NAB2	p429	wild type NAB2	Tudek et al., 2018
ΔNTD	p437	[4-97 deleted], C-terminal flag-tag	This study
ΔQQQP	p433	[104-169 deleted]	This study

ΔTPS	p441	[170-200 deleted], C-terminal flag-tag	This study
ΔRGG	p442	[201-255 deleted]; SV40 NLS-GlyGly inserted at the N-terminus, C-terminal flag-tag	This study
ΔZnF1-4	p440	[262-389 deleted], C-terminal flag-tag	This study
ΔZnF5-7-CT	p432	[395-525 deleted]	This study
ΔCT	p450	[486-525 deleted]	This study
ΔZnF1	p443	[262-278 deleted], C-terminal flag-tag	This study
ΔZnF2	p444	[283-300 deleted], C-terminal flag-tag	This study
ΔZnF3	p446	[340-355 deleted], C-terminal flag-tag	This study
ΔZnF4	p447	[371-386 deleted], C-terminal flag-tag	This study
ΔL2-3	p445	[305-333 deleted], C-terminal flag-tag	This study
ΔL4-5	p448	[391-408 deleted], C-terminal flag-tag	This study
NAB2-flag (wild type)	p434	C-terminal flag-tag	This study
H434D, N466D	p482	H434D,N466D	This study
F450A	p495	F450A	This study
F353A	p517	F353A	This study
F353A, H434D, N466D	p518	F353A,H434D,N466D	This study
F353A, F450A	p508	F353A,F450A	This study
K341A, F342A	p516	K341A,F342A	This study
K341A, F342A, F450A	p507	K341A,F342A,F450A	This study
H420D	p478	H420D	This study
H420D, H434D, N466D	p496	H420D,H434D,N466D	This study
H434D, N466D, I467D, Y468D	p497	H434D,N466D,I467D,Y468D	This study
ZnF1-7-CT >> Hsa ZnF1-5	p458	SceNab2 amino acids 1-255 fused to HsaZC3H14 amino acids 594-736	This study
**p(*NAB2/CEN, LEU2*) plasmid****			
empty	p513H	p(*CEN, LEU2*); empty plasmid	This study
NAB2	p519	wild type NAB2	This study
N466D,H434D	p525	N466D,H434D	This study
F450A	p528	F450A	This study
F353A	p530	F353A	This study
K341A,F342A	p529	K341A,F342A	This study
N466D,H434D,F353A	p537	N466D,H434D,F353A	This study
F450A,F353A	p535	F450A,F353A	This study
F450A,K341A,F342A	p534	F450A,K341A,F342A	This study
NAB2 (wild type)	pMT105	A26>A11 (autoregulatory downstream sequence)	This study
NAB2 (wild type)	pMT106	A26>A16 (autoregulatory downstream sequence)	This study
NAB2 (wild type)	pAV002	A26>A21 (autoregulatory downstream sequence, spontaneous mutation in p513 clone F)	This study
NAB2 (wild type)	pMT107	A26>A33 (autoregulatory downstream sequence)	This study
NAB2 (wild type)	pMT108	A26>A42 (autoregulatory downstream sequence)	This study
**pESC-URA 2µ, ori(f1), ori(pUC), URA3 , Ampr, TCYC1, MCS, PGAL1, PGAL10, MCS, TADH1; NAB2-genes cloned under PGAL10 with EcoRI and NotI*			
***pRSII415 LEU2, CEN6/ARSH4, pBluescript II SK+ NAB2 promoter (400 bp)_ EcoRI-NotI_NAB2 downstream sequence (400 bp); NAB2 genes cloned with EcoRI and NotI*			

Supplemental Table S3. Primers

	Plasmid number	Sequence [deletions], point mutations, modifications	Two-step PCR	PCR template	Primer sequence (5'-3')	Primer Description
NAB2* in pPgal plasmid						
ΔNTD	p437	[4-97], C-terminal flag-tag	n.a.	p429	ACAGCAGAATTCATGTCTCAAAGCTTGGGACAATCGGATATC	Forw_EcoRI-[MSQ-Nab2 delta NTD 4-97]
					ACAAACGCGGCCGCGTTCATTTCCGTATCTTGTTCTTG	Rev NotI-[C-term of Nab2], no term codon
ΔQQQP	p433	[104-169]	n.a.	pAC1039	gtccactgGAATTCATGTCTCAAGAACAGTACACAGAAAA	Fwd for Nab2 ORF amplification with EcoRI site
					gctgacctGCGGCCGCTCAGTTCATTTCCGTATCTTGTTC	Rev for Nab2 ORF amplification with NotI site
ΔTPS	p441	[170-200], C-terminal flag-tag	first step, N	p429	ATGTCTCAAGAACAGTACACAG	Forw Nab2 N-term
					GGCTGTTATCATTCTGGGTCCCTAGTTGT	delTPS (rev A)
			first step, C	p429	GACCCAGAATGATAACAGCCAAAGGTTCAC	delTPS (fw B)
					GTTCATTTCCGTATCTTGTTCTTG	Rev Nab2 C-term
			second step	N+C (from first step)	gtccactgGAATTCATGTCTCAAGAACAGTACACAGAAAA	Fwd for Nab2 ORF amplification with EcoRI site
					ACAAACGCGGCCGCGTTCATTTCCGTATCTTGTTCTTG	Rev NotI-[C-term of Nab2], no term codon, can be cloned in frame with FLAG in pESC
ΔRGG	p442	[201-255]; SV40 NLS-GlyGly inserted at the N-terminus, C-terminal flag-tag	first step, N	p429	ATGTCTCAAGAACAGTACACAG	Forw Nab2 N-term
					CTTTCTTGGTTACAGGCGCAAACTGAGG	delRGG-ver2 (rev A)
			first step, C	p429	TGCGCCTGTAACCAAGAAAGAGGGGCGT	delRGG-ver2 (fw B)
					GTTCATTTCCGTATCTTGTTCTTG	Rev Nab2 C-term
			second step	N+C (from first step)	ACAGCAGAATTCATGCCAAAAAAAAAAAGAAAAGTTGGCGGCTCTCAAGAACAGTACACAGAAAAC	Forw_EcoRI-[N-term of SV40*NLS-GG-Nab2 N-term]
					ACAAACGCGGCCGCGTTCATTTCCGTATCTTGTTCTTG	Rev NotI-[C-term of Nab2], no term codon, can be cloned in frame with FLAG in pESC
ΔZnF1-4	p440	[262-389], C-terminal flag-tag	first step, N	p429	ATGTCTCAAGAACAGTACACAG	Forw Nab2 N-term
					TGATCTTCGAACGCCCCTCTTTCTTGGTTG	Rev Nab2 residue ...261+10 bp overhang complementary to residues 390… -->2-step PCR deletion of ZnF1-4
			first step, C	p429	AGAGGGGCGTTCGAAGATCAAGGAAGTAAAACC	Forw Nab2 residue 390…+10 bp overhang complementary to residues …261 -->2-step PCR deletion of ZnF1-4
					GTTCATTTCCGTATCTTGTTCTTG	Rev Nab2 C-term
			second step	N+C (from first step)	gtccactgGAATTCATGTCTCAAGAACAGTACACAGAAAA	Fwd for Nab2 ORF amplification with EcoRI site
					ACAAACGCGGCCGCGTTCATTTCCGTATCTTGTTCTTG	Rev NotI-[C-term of Nab2], no term codon, can be cloned in frame with FLAG in pESC
ΔZnF5-7-CT	p432	[395-525]	n.a.	p429	gtccactgGAATTCATGTCTCAAGAACAGTACACAGAAAA	Fwd for Nab2 ORF amplification with EcoRI site
					ACAACAGCGGCCGCTCACAATGAAGAGTGGGCCTTTC	Rev_Truncation of Nab2 ZnF 5-7, residues 390-525. Introduce termination codon and NotI site
ΔCT	p450	[486-525]	n.a.	p429	gtccactgGAATTCATGTCTCAAGAACAGTACACAGAAAA	Fwd for Nab2 ORF amplification with EcoRI site
					ACAAACGCGGCCGCTCAAGCGCCTTTCTTTTCCG	delCT[486-525]-NotI-without flag_rev
ΔZnF1	p443	[262-278], C-terminal flag-tag	first step, N	p429	ATGTCTCAAGAACAGTACACAG	Forw Nab2 N-term
					CCTTAGTTGGACGCCCCTCTTTCTTGGT	delZnF1 (rev A)
			first step, C	p429	AGAGGGGCGTCCAACTAAGGTATGTAATGAATATCC	delZnF1 (fw B)
					GTTCATTTCCGTATCTTGTTCTTG	Rev Nab2 C-term
			second step	N+C (from first step)	gtccactgGAATTCATGTCTCAAGAACAGTACACAGAAAA	Fwd for Nab2 ORF amplification with EcoRI site
					ACAAACGCGGCCGCGTTCATTTCCGTATCTTGTTCTTG	Rev NotI-[C-term of Nab2], no term codon, can be cloned in frame with FLAG in pESC
ΔZnF2	p444	[283-300], C-terminal flag-tag	first step, N	p429	ATGTCTCAAGAACAGTACACAG	Forw Nab2 N-term
					CTTCATTTGGTACCTTAGTTGGGTGTGCATG	delZnF2 (rev A)
			first step, C	p429	CCCAACTAAGGTACCAAATGAAGATGAAGAGTTGATG	delZnF2 (fw B)
					GTTCATTTCCGTATCTTGTTCTTG	Rev Nab2 C-term
			second step	N+C (from first step)	gtccactgGAATTCATGTCTCAAGAACAGTACACAGAAAA	Fwd for Nab2 ORF amplification with EcoRI site

					ACAAACGCGGCCGCGTTCATTTCCGTATCTTGTTCTTG	Rev NotI-[C-term of Nab2], no term codon, can be cloned in frame with FLAG in pESC
ΔZnF3	p446	[340-355], C-terminal flag-tag	first step, N	p429	ATGTCTCAAGAACAGTACACAG	Forw Nab2 N-term
					CTGGTGTTGGCAGAACGATACCAGTTTGTACC	delZnF3 (rev A)
			first step, C	p429	TATCGTTCTGCCAACACCAGCAAATGAAG	delZnF3 (fw B)
					GTTCATTTCCGTATCTTGTTCTTG	Rev Nab2 C-term
			second step	N+C (from first step)	gtccactgGAATTCATGTCTCAAGAACAGTACACAGAAAA	Fwd for Nab2 ORF amplification with EcoRI site
					ACAAACGCGGCCGCGTTCATTTCCGTATCTTGTTCTTG	Rev NotI-[C-term of Nab2], no term codon, can be cloned in frame with FLAG in pESC
ΔZnF4	p447	[371-386], C-terminal flag-tag	first step, N	p429	ATGTCTCAAGAACAGTACACAG	Forw Nab2 N-term
					ACAATGAAGACCACATTAGATCAATGACTTTCG	delZnF4 (rev A)
			first step, C	p429	ATTGATCTAATGTGGTCTTCATTGTCGAAGATCAAGG	delZnF4 (fw B)
					GTTCATTTCCGTATCTTGTTCTTG	Rev Nab2 C-term
			second step	N+C (from first step)	gtccactgGAATTCATGTCTCAAGAACAGTACACAGAAAA	Fwd for Nab2 ORF amplification with EcoRI site
					ACAAACGCGGCCGCGTTCATTTCCGTATCTTGTTCTTG	Rev NotI-[C-term of Nab2], no term codon, can be cloned in frame with FLAG in pESC
ΔL2-3	p445	[305-333], C-terminal flag-tag	first step, N	p429	ATGTCTCAAGAACAGTACACAG	Forw Nab2 N-term
					CAGTTTGTACATCTTCATTTGGATGTAAAAACTCAC	delLinker2-3 (rev A)
			first step, C	p429	CATCCAAATGAAGATGTACAAACTGGTATCGTTCTG	delLinker2-3 (fw B)
					GTTCATTTCCGTATCTTGTTCTTG	Rev Nab2 C-term
			second step	N+C (from first step)	gtccactgGAATTCATGTCTCAAGAACAGTACACAGAAAA	Fwd for Nab2 ORF amplification with EcoRI site
					ACAAACGCGGCCGCGTTCATTTCCGTATCTTGTTCTTG	Rev NotI-[C-term of Nab2], no term codon, can be cloned in frame with FLAG in pESC
ΔL4-5	p448	[391-408], C-terminal flag-tag	first step, N	p429	ATGTCTCAAGAACAGTACACAG	Forw Nab2 N-term
					AGGACTTTTCCGACAATGAAGAGTGGGC	delLinker4-5 (rev A)
			first step, C	p429	CTTCATTGTCGGAAAAGTCCTTAGAACAATGTAAG	delLinker4-5 (fw B)
					GTTCATTTCCGTATCTTGTTCTTG	Rev Nab2 C-term
			second step	N+C (from first step)	gtccactgGAATTCATGTCTCAAGAACAGTACACAGAAAA	Fwd for Nab2 ORF amplification with EcoRI site
					ACAAACGCGGCCGCGTTCATTTCCGTATCTTGTTCTTG	Rev NotI-[C-term of Nab2], no term codon, can be cloned in frame with FLAG in pESC
NAB2-flag (wild type)	p434	C-terminal flag-tag	n.a.	p429	gtccactgGAATTCATGTCTCAAGAACAGTACACAGAAAA	Fwd for Nab2 ORF amplification with EcoRI site
					ACAAACGCGGCCGCGTTCATTTCCGTATCTTGTTCTTG	Rev NotI-[C-term of Nab2], no term codon, can be cloned in frame with FLAG in pESC
H434D	p481	H434D	n.a.	p429	TTCACGGCACATAATATCAGAACGAGCATGTCT	H434D-NAB2_fw
					GGAGCAAACTGTACTAGAATTGA	H434X-NAB2_rev
H434D, N466D	p482	H434D, N466D	n.a.	p481	TCTGAATAGACAGTAAATATCCTTACAATTGACACC	N466D-NAB2_fw
					CATCCTCCAGGCAGAGTAC	466-468-NAB2_rev
F450A	p495	F450A	n.a.	p429	CATTAATTGGATGGCCAGCTAAACAATCAATTC	nab2-F450A fw
					AAGATTGTAGATTTGGTGTCAATTG	nab2_F450A_rev
F353A	p517	F353A	n.a.	p429	GCTGGTGTTGGATGACCAGCTGGGCATGATGGATTGGA	F353A_fw
					AAATGAAGATGCGAAAGTCATTG	353_rev
F353A, H434D, N466D	p518	F353A, H434D, N466D	n.a.	p482	GCTGGTGTTGGATGACCAGCTGGGCATGATGGATTGGA	F353A_fw
					AAATGAAGATGCGAAAGTCATTG	353_rev
F353A, F450A	p508	F353A, F450A	n.a.	p495	GCTGGTGTTGGATGACCAGCTGGGCATGATGGATTGGA	F353A_fw
					AAATGAAGATGCGAAAGTCATTG	353_rev
K341A, F342A	p516	K341A, F342A	n.a.	p429	GAACACAGAGCCCCAGCAGCACACAGAACGATACCAGTTTG	K341A,F342A_fw
					CAATCCATCATGCCCATTTG	342_rev
K341A, F342A, F450A	p507	K341A, F342A, F450A	n.a.	p495	GAACACAGAGCCCCAGCAGCACACAGAACGATACCAGTTTG	K341A,F342A_fw
					CAATCCATCATGCCCATTTG	342_rev
H420D	p478	H420D	n.a.	p429	ACGTTTATTGGTGCAATCCGTACCGAACTTACA	H420D-NAB2_fw
					TGCAAATATAGACATGCTCGTTC	H420D-NAB2_rev
H420D, H434D, N466D	p496	H420D, H434D, N466D	n.a.	p482	ACGTTTATTGGTGCAATCCGTACCGAACTTACA	H420D-NAB2_fw
					TGCAAATATAGACATGCTCGTTC	H420D-NAB2_rev

H434D, N466D, I467D, Y468D	p497	H434D, N466D, I467D, Y468D	n.a.	p482	ATCATCATCCTTACAATTGACACCAAATC	I467D,Y468D on N466D_fw
					TGTCTATTCAGACATCCTCCAG	468-rev
ZnF1-7-CT >> Hsa ZnF1-5	p458	SceNab2 amino acids 1-255 fused to HsaZC3H14 amino acids 594-736	first step, N	p429	ATGTCTCAAGAACAGTACACAG	Forw Nab2 N-term
					TGGAGTGAAGTTCATATTACTCTC	NAB2-255 rev
			second step	N (from first step) + GeneArt DNA fragment	gtccactgGAATTCATGTCTCAAGAACAGTACACAGAAAA	Fwd for Nab2 ORF amplification with EcoRI site
					ACAAACGCGGCCGCTCATTCGGAGGTTTGTGGTC	ZC3H14 Cterm rev-NotI
**p(*CEN/LEU2*) parent plasmids**						
empty	p513	n.a.	n.a.	pAC1039	cctcgaggtcgacggtatcgataagcttgCGAGACGTTTATATAGGGATGTG	NAB2-promoter_fw
					gcggccgccccgggctgcaggaattcgTTCTGATGTACTTCCACTTCCT	NAB2-promoter_rev
			n.a.	pAC1039	cgaattcctgcagcccggggcggccgcTTACTATTAAAATCACGGAACGAAATTC	NAB2-terminator_fw
					taaagggaacaaaagctggagctccTGATTGAAACCCAGTCTGTCC	NAB2_terminator_rev
empty	pMT101	autoregulatory A26>A11	n.a.	p513H	GTTTTTAAACAGTTCCTGATC	NAB2-UTR_fw
					TTTTTTTTTTTAAAATCTAAATATGTTGCTTG	NAB2-UTR_A11_rev
empty	pMT102	autoregulatory A26>A16	n.a.	p513H	GTTTTTAAACAGTTCCTGATC	NAB2-UTR_fw
					TTTTTTTTTTTTTTTTAAAATCTAAATATGTTGCTTG	NAB2-UTR_A16_rev
empty	pMT103	autoregulatory A26>A33	n.a.	p513H	GTTTTTAAACAGTTCCTGATC	NAB2-UTR_fw
					TTTTTTTTTTTTTTTTTTTTTTTTTTTTTTTTTAAAATCTAAATATGTTGCTTG	NAB2-UTR_A33_rev
empty	pMT104	autoregulatory A26>A42	n.a.	p513H	GTTTTTAAACAGTTCCTGATC	NAB2-UTR_fw
					TTTTTTTTTTTTTTTTTTTTTTTTTTTTTTTTTTTTTTTTTTAAAATCTAAATATGTTGCTTG	NAB2-UTR_A42_rev
***E. coli* expression plasmids**						
Nab2	pEG005	pET28b C-terminal 6xHis	n.a.	p429	CTTTAAGAAGGAGATATACCATGTCTCAAGAACAGTACACAG	Nab2-Nterm_homol to pET28b_Gibson
					TGGTGCTCGAGTGCGGCCGCTCAATGATGATGATGATGATGGTTCATTTCCGTATCTTGTT	PRIM047_Nab2-Cterm-6His_homol to pET28b_Gibson
Nab2-FKBP12	pEG025	pET28b C-terminal FKBP12 (H. sapiens FKBP12 residues 1-107), C-terminal 6xHis	n.a.	p429	CTTTAAGAAGGAGATATACCATGTCTCAAGAACAGTACACAG	Nab2-Nterm_homol to pET28b_Gibson
					GTTCATTTCCGTATCTTGTT	NAB2 ORF rev
			n.a.	genomic DNA of HHY212 (Euroscarf)	GATACGGAAATGAACGGAGTGCAGGTGGAAACCATCTC	NAB2-3end_FKBP12_fw
					CTCGAGTGCGGCCGCTCAATGATGATGATGATGATGGCCAGTTTCCAGTTTTAGAAGCTC	FKBP12-6His_rev_pET28 overhand
Nab2-FRB	pEG026	pET28b C-terminal FRB (H. sapiens mTOR residues 2021-2113, T2098L), C-terminal 6xHis	n.a.	p429	CTTTAAGAAGGAGATATACCATGTCTCAAGAACAGTACACAG	Nab2-Nterm_homol to pET28b_Gibson
					GTTCATTTCCGTATCTTGTT	NAB2 ORF rev
			n.a.	pFA6a-FRB-GFP-KanMX6 (Euroscarf P30580)	AACAAGATACGGAAATGAACATCCTCTGGCATGAGATG	NAB2_3end_overhang_FRB-fw
					CTCGAGTGCGGCCGCTCAATGATGATGATGATGATGGCCCTTTGAGATTCGTCGGA	FRB-G-6his-stop-pET28b_rev
Nab2_ΔRGG	pEG007	pET28b [201-255], C-terminal 6xHis	n.a.	p442	CTTTAAGAAGGAGATATACCATGTCTCAAGAACAGTACACAG	Nab2-Nterm_homol to pET28b_Gibson
					TGGTGCTCGAGTGCGGCCGCTCAATGATGATGATGATGATGGTTCATTTCCGTATCTTGTT	PRIM047_Nab2-Cterm-6His_homol to pET28b_Gibson
Nab2_ΔZnF1-4	pEG006	pET28b [262-389], C-terminal 6xHis	n.a.	p440	CTTTAAGAAGGAGATATACCATGTCTCAAGAACAGTACACAG	Nab2-Nterm_homol to pET28b_Gibson
					TGGTGCTCGAGTGCGGCCGCTCAATGATGATGATGATGATGGTTCATTTCCGTATCTTGTT	PRIM047_Nab2-Cterm-6His_homol to pET28b_Gibson
Nab2_ΔZnF5-7	pEG012	pET28b [390-485], C-terminal 6xHis	n.a.	pEG005	GCACCCAATTCAAACGTT	deltaZnF567_fw
					CAATGAAGAGTGGGCCTT	deltaZnF567_rev
Nab2_N466D,H434D	pEG008	pET28b N466D,H434D, C-terminal 6xHis	n.a.	p482	CTTTAAGAAGGAGATATACCATGTCTCAAGAACAGTACACAG	Nab2-Nterm_homol to pET28b_Gibson
					TGGTGCTCGAGTGCGGCCGCTCAATGATGATGATGATGATGGTTCATTTCCGTATCTTGTT	PRIM047_Nab2-Cterm-6His_homol to pET28b_Gibson
Nab2_F450A	pEG009	pET28b F450A, C-terminal 6xHis	n.a.	p495	CTTTAAGAAGGAGATATACCATGTCTCAAGAACAGTACACAG	Nab2-Nterm_homol to pET28b_Gibson

					TGGTGCTCGAGTGCGGCCGCTCAATGATGATGATGATGATGGTTCATTTCCGTATCTTGTT	PRIM047_Nab2-Cterm-6His_homol to pET28b_Gibson
Nab2_F353A	pEG010	pET28b F353A, C-terminal 6xHis	n.a.	p517	CTTTAAGAAGGAGATATACCATGTCTCAAGAACAGTACACAG	Nab2-Nterm_homol to pET28b_Gibson
					TGGTGCTCGAGTGCGGCCGCTCAATGATGATGATGATGATGGTTCATTTCCGTATCTTGTT	PRIM047_Nab2-Cterm-6His_homol to pET28b_Gibson
Nab2_F353A,H434D,N466D	pEG017	pET28b F353A,H434D,N466D, C-terminal 6xHis	n.a.	p518	CTTTAAGAAGGAGATATACCATGTCTCAAGAACAGTACACAG	Nab2-Nterm_homol to pET28b_Gibson
					TGGTGCTCGAGTGCGGCCGCTCAATGATGATGATGATGATGGTTCATTTCCGTATCTTGTT	PRIM047_Nab2-Cterm-6His_homol to pET28b_Gibson
Nab2_F353A,F450A	pEG011	pET28b F353A,F450A, C-terminal 6xHis	n.a.	p508	CTTTAAGAAGGAGATATACCATGTCTCAAGAACAGTACACAG	Nab2-Nterm_homol to pET28b_Gibson
					TGGTGCTCGAGTGCGGCCGCTCAATGATGATGATGATGATGGTTCATTTCCGTATCTTGTT	PRIM047_Nab2-Cterm-6His_homol to pET28b_Gibson

Supplemental Table S4. Oligonucleotides

**Identifier**	**Name of RNA oligonucleotide**	**Sequence 5'-3' (underlined are deoxyribonucleotides)**	**5' mod**	**3' mod**	**Sequence reference**	**Manufactured by**
R001	*CYC142*	UUUAUAGUUAUGUUAGUAUUAAGAACGUUAUUUAUAUUUCAA	Atto680	-	Schmid *et al.* 2012	Eurogentec
R022	*CYC159*	UUUAUAGUUAUGUUAGUAUUAAGAACGUUAUUUAUAUUUCAAAUUUUUCUUUUUUUUCU	Atto680	Cy3	*CYC1a* (Hill *et al.* 2019) extended by 3 nts at the 3'end	Splinted ligation of R001 and R021 with D004; 1:1.2:1; T4 DNA Ligase 0.4 U/µL
R021	*dwCYC117*	AUUUUUCUUUUUUUUCU	Phosphate	Cy3	This study	Eurogentec
R003	*A59*	AAAAAAAAAAAAAAAAAAAAAAAAAAAAAAAAAAAAAAAAAAAAAAAAAAAAAAAAAAA	Atto680	-	This study	Eurogentec
R006	*A59*	AAAAAAAAAAAAAAAAAAAAAAAAAAAAAAAAAAAAAAAAAAAAAAAAAAAAAAAAAAA	Phosphate	-	This study	Eurogentec
R002	*A30*	AAAAAAAAAAAAAAAAAAAAAAAAAAAAAA	Phosphate	-	This study	Eurogentec
R007	*A15*	AAAAAAAAAAAAAAA	Atto680	-	This study	Eurogentec
R017	*A20*	AAAAAAAAAAAAAAAAAAAA	Atto680	-	This study	Splinted ligation of R007 and R008 with D002, 1:1.5:1.5, T4 DNA Ligase 0.4 U/µL
R012	*A25*	AAAAAAAAAAAAAAAAAAAAAAAAA	Atto680	-	This study	Splinted ligation of R007 and R009 + R010 with D002, 1:1.5:1, T4 DNA Ligase 0.4 U/µL
R013	*A30*	AAAAAAAAAAAAAAAAAAAAAAAAAAAAAA	Atto680	-	This study	Splinted ligation of R007 and R009 + R010 with D002, 1:1.5:1, T4 DNA Ligase 0.4 U/µL
R014	*A35*	AAAAAAAAAAAAAAAAAAAAAAAAAAAAAAAAAAA	Atto680	-	This study	Splinted ligation of R007 and R009 + R010 with D002, 1:1.5:1, T4 DNA Ligase 0.4 U/µL
R016	*A45*	AAAAAAAAAAAAAAAAAAAAAAAAAAAAAAAAAAAAAAAAAAAAA	Atto680	-	This study	Splinted ligation of R007 and R009 + R010 with D002, 1:1.5:1, T4 DNA Ligase 0.4 U/µL
R008	*A5*	AAAAA	Phosphate	-	This study	Eurogentec
R009	*A10*	AAAAAAAAAA	Phosphate	-	This study	Eurogentec
R010	*A15*	AAAAAAAAAAAAAAA	Phosphate	-	This study	Eurogentec
D002	splint DNA	TTTTTTTTTTTTTTTTTTTTTTTTTTTTTTTTTTTTTTTTTTTTTTTTTTGAAATATAAATAA	-	-	This study	Merck/SigmaAldrich
D004	splint DNA	AAAAGAAAAATTTGAAATATAAA	-	-	This study	Merck/SigmaAldrich
DL163	*HSP104* RNaseH targeting oligo	ACATTTTCATCACGAGATTTACCC	-	-	Libri *et al.* 2002	Merck/SigmaAldrich
DL164	*HSP104* 3´hybridization DNA probe	TTATCGTCATCACCTAACGTGTCAGCCCCTATAGTAGCTTCGTGATTTGGTAGAACTTCC	-	-	Libri *et al.* 2002	Merck/SigmaAldrich
MS618	*SCR1* hybridization DNA probe	GGTCACCTTTGCTGACGCTGG	-	-	Schmid *et al.* 2015	Merck/SigmaAldrich
NL-A48	nanolever green channel (switchSENSE)	TAGTGCTGTAGGAGAATATACGGGCTGCTCGTGTTGACAAGTACTGAT	-	green dye	Dynamic Biosensors	n.a.
cNLA-X2	nanolever green channel (switchSENSE)	ACAGATGACTGCTCGTCAATATCAGTACTTGTCAACACGAGCAGCCCGTATATTCTCCTACAGCACTA	-	-	This study	Eurogentec
CX2	DNA only (switchSENSE)	ATTGACGAGCAGTCATCTGT	-	-	This study	Eurogentec
NL-B48	nanolever red channel (switchSENSE)	TAGTCGTAAGCTGATATGGCTGATTAGTCGGAAGCATCGAACGCTGAT	-	red dye	Dynamic Biosensors	n.a.

cNLB-X1	nanolever red channel (switchSENSE)	ACACTACTGACGAGCACAATATCAGCGTTCGATGCTTCCGACTAATCAGCCATATCAGCTTACGACTA	-	-	This study	Eurogentec
CX1-rA60	rA60 (switchSENSE)	ATTGTGCTCGTCAGTAGTGTAAAAAAAAAAAAAAAAAAAAAAAAAAAAAAAAAAAAAAAAAAAAAAAAAAAAAAAAAAAA	-	-	This study	Eurogentec
CX1-rA30	rA30 (switchSENSE)	ATTGTGCTCGTCAGTAGTGTAAAAAAAAAAAAAAAAAAAAAAAAAAAAAA	-	-	This study	Eurogentec

Supplemental Table S5. Polyadenylation reaction conditions.

Figure panel	Pre-incubation step 1	Pre-incubation step 2	Final concentrations					
			RNA	CPF	CF IA	CF IB	Nab2-6xHis	ATP
1B & 1D	RNA +CF IA +CF IB (5 min)	+CPF (3 min)	50 nM	50 nM	225 nM	225 nM	-	2 mM
S1A	RNA + CPF (5 min)	-	100, 50, 25 nM	100, 50, 25 nM	500 nM	500 nM	-	2 mM
S1B	RNA +CF IA +CF IB (5 min)	-	25 nM	75 nM	500 nM	500 nM	-	2 mM
S1C	RNA +CF IA +CF IB (5 min)	-	25 nM	75 nM	500 nM	500 nM	-	100 µM (3'dATP)
2A	RNA +CF IA +CF IB (5 min)	+CPF -/+Nab2 (3 min)	25 nM	75 nM	500 nM	500 nM	0, 500 nM	2 mM
2B	RNA +CF IA +CF IB (5 min)	+CPF -/+Nab2 (4 min)	25 nM	75 nM	500 nM	500 nM	0, 50, 100, 175, 250, 350, 500, 750, 1000 nM	2 mM
2C	RNA +CF IA +CF IB (5 min)	+CPF -/+Nab2 (4 min)	25 nM	75 nM	500 nM	500 nM	175, 350 nM	500, 100, 50, 20, 10, 5 µM
4C&S4B	RNA +CF IA +CF IB (6 min)	+CPF -/+Nab2 -/+ Rapamycin or DMSO (5 min)	25 nM	75 nM	500 nM	500 nM	0, 175, 500, 1000 nM	2 mM
5C	RNA +CF IA +CF IB (5 min)	+CPF -/+Nab2 (3 min)	25 nM	75 nM	500 nM	500 nM	0, 175, 250, 350, 500, 750, 1000 nM	2 mM
6B	RNA +CF IA +CF IB (5 min)	+CPF -/+Nab2 (3 min)	25 nM	75 nM	500 nM	500 nM	0, 175, 250, 350, 500, 750, 1000 nM	2 mM
S6B	RNA +CF IA +CF IB (5 min)	+CPF -/+Nab2 (3 min)	25 nM	75 nM	500 nM	500 nM	0,1000 nM	2 mM

REFERENCES FOR SUPPLEMENTAL MATERIAL

Aibara S, Gordon JMB, Riesterer AS, Mclaughlin SH, Stewart M. 2017. Structural basis for the dimerization of Nab2 generated by RNA binding provides insight into its contribution to both poly(A) tail length determination and transcript compaction in Saccharomyces cerevisiae. *Nucleic Acids Res* **45**: 1529–1538.

Anderson JT, Wilson SM, Datar K V, Swanson MS. 1993. NAB2: a Yeast Nuclear Polyadenylated RNA-Binding Protein Essential for Cell Viability. *Mol Cell Biol* **13**: 2730–2741.

Bieniossek C, Richmond TJ, Berger I. 2008. MultiBac: Multigene baculovirus-based eukaryotic protein complex production. *Curr Protoc Protein Sci* **5.20**: 1–26.

Casañal A, Kumar A, Hill CH, Easter AD, Emsley P, Degliesposti G, Gordiyenko Y, Santhanam B, Wolf J, Wiederhold K, et al. 2017. Architecture of eukaryotic mRNA 3′-end processing machinery. *Science* **358**: 1056–1059.

Chee MK, Haase SB. 2012. New and Redesigned pRS Plasmid Shuttle Vectors for Genetic Manipulation of *Saccharomyces cerevisiae*. *G3 Genes|Genomes|Genetics* **2**: 515–526.

Hill CH, Boreikaitė V, Kumar A, Casañal A, Kubík P, Degliesposti G, Maslen S, Mariani A, von Loeffelholz O, Girbig M, et al. 2019. Activation of the Endonuclease that Defines mRNA 3′ Ends Requires Incorporation into an 8-Subunit Core Cleavage and Polyadenylation Factor Complex. *Mol Cell* **73**: 1217-1231.e11.

Kumar A, Yu CWH, Rodríguez-Molina JB, Li XH, Freund SMV, Passmore LA. 2021. Dynamics in Fip1 regulate eukaryotic mRNA 3′ end processing. *Genes Dev* **35**: 1510–1526.

Libri D, Dower K, Boulay J, Thomsen R, Rosbash M, Jensen TH. 2002. Interactions between mRNA Export Commitment, 3′-End Quality Control, and Nuclear Degradation. *Mol Cell Biol* **22**: 8254–8266.

Marfatia KA, Crafton EB, Green DM, Corbett AH. 2003. Domain Analysis of the Saccharomyces cerevisiae Heterogeneous Nuclear Ribonucleoprotein, Nab2p. *J Biol Chem* **278**: 6731–6740.

Martínez-Lumbreras S, Santiveri CM, Mirassou Y, Zorrilla S, Pérez-Cañadillas JM. 2013. Two singular types of CCCH tandem zinc finger in Nab2p contribute to polyadenosine RNA recognition. *Structure* **21**: 1800–1811.

Rodríguez-Molina JB, O’Reilly FJ, Fagarasan H, Sheekey E, Maslen S, Skehel JM, Rappsilber J, Passmore LA. 2022. Mpe1 senses the binding of pre-mRNA and controls 3′ end processing by CPF. *Mol Cell* **82**: 2490-2504.e12.

Schmid M, Olszewski P, Pelechano V, Gupta I, Steinmetz LM, Jensen TH. 2015. The Nuclear PolyA-Binding Protein Nab2p Is Essential for mRNA Production. *Cell Rep* **12**: 128–139.

Schmid M, Poulsen MB, Olszewski P, Pelechano V, Saguez C, Gupta I, Steinmetz LM, Moore C, Jensen TH. 2012. Rrp6p Controls mRNA Poly(A) Tail Length and Its Decoration with Poly(A) Binding Proteins. *Mol Cell* **47**: 267–280.

Tudek A, Schmid M, Makaras M, Barrass JD, Beggs JD, Jensen TH. 2018. A Nuclear Export Block Triggers the Decay of Newly Synthesized Polyadenylated RNA. *Cell Rep* **24**: 2457–2467.

Turtola M, Manav CM, Kumar A, Tudek A, Mroczek S, Krawczyk PS, Dziembowski A, Schmid M, Passmore LA, Casanal A, et al. 2021. Three-layered control of mRNA poly(A) tail synthesis in Saccharomyces cerevisiae. *Genes Dev* **35**: 1290–1303.
